# Understanding, engineering, and modulating the growth of neural networks: An interdisciplinary approach

**DOI:** 10.1063/5.0043014

**Published:** 2021-06-17

**Authors:** Vidur Raj, Chennupati Jagadish, Vini Gautam

**Affiliations:** 1 Department of Electronic Materials Engineering, Research School of Physics, The Australian National University, Canberra, Australian Capital Territory 2601, Australia; 2 ARC Centre of Excellence for Transformative Meta-Optical Systems, Research School of Physics, The Australian National University, Canberra, Australian Capital Territory 2601, Australia; 3 Department of Biomedical Engineering, Faculty of Engineering and Information Technology, The University of Melbourne, Melbourne, Victoria 3010, Australia

## Abstract

A deeper understanding of the brain and its function remains one of the most significant scientific challenges. It not only is required to find cures for a plethora of brain-related diseases and injuries but also opens up possibilities for achieving technological wonders, such as brain–machine interface and highly energy-efficient computing devices. Central to the brain's function is its basic functioning unit (i.e., the neuron). There has been a tremendous effort to understand the underlying mechanisms of neuronal growth on both biochemical and biophysical levels. In the past decade, this increased understanding has led to the possibility of controlling and modulating neuronal growth *in vitro* through external chemical and physical methods. We provide a detailed overview of the most fundamental aspects of neuronal growth and discuss how researchers are using interdisciplinary ideas to engineer neuronal networks *in vitro*. We first discuss the biochemical and biophysical mechanisms of neuronal growth as we stress the fact that the biochemical or biophysical processes during neuronal growth are not independent of each other but, rather, are complementary. Next, we discuss how utilizing these fundamental mechanisms can enable control over neuronal growth for advanced neuroengineering and biomedical applications. At the end of this review, we discuss some of the open questions and our perspectives on the challenges and possibilities related to controlling and engineering the growth of neuronal networks, specifically in relation to the materials, substrates, model systems, modulation techniques, data science, and artificial intelligence.

## I. INTRODUCTION

The brain is undoubtedly one of the most sophisticated organs and an unparalleled energy-efficient computing machine that can perform, process, and relay information much more efficiently compared to even the best supercomputer built by humans.^1–3^ However, the brain is highly complex as well, and because of its complexity, the current understanding of the brain and brain-related diseases remains limited. Central to the brain's function are neurons and neuronal networks, and unraveling the basic working principles of neurons and neural computing not only is required to gain an in-depth understanding of brain-related diseases and their cure but also may provide the key for the development of highly power-efficient computing devices, brain reverse engineering, and brain–machine interfaces (BMIs). At present, *in vitro* neuronal cell cultures remain one of the most accessible and economic surrogates to study some fundamental processes behind the complex brain.

In earlier days, *in vitro* neuronal cultures were mainly used to understand the biochemical mechanisms involved in neuron growth and connections. The advent of new technological advancements, such as micro-electrode arrays (MEAs),^4–6^ liquid AFM,^7,8^ and two-/three-photon microscopies,^9–11^ of *in vitro* neuronal cultures now facilitates the study of both biochemical and biophysical aspects of studying neuronal growth. Additionally, a significant breakthrough in *in vitro* neuronal culture has been the advancement in stem cell technology, which has allowed the direct study of human neurons.^12^ In recent years, researchers have even been able to develop *in vitro* 3D human brain models (brain organoids) to replicate some of the critical multicellular, anatomical, and even functional hallmarks of entire regions of the brain at the micrometer to millimeter scale.^13–18^ Furthermore, advances in micro- and nanofabrication techniques have helped to grow neurons in artificial matrices in a controlled and precise manner to envision the possibility of BMI and brain-on-chip technologies.

Central to *in vitro* neuronal growth is the ability and efficacy to which neurons can be controlled or manipulated as they grow, and this has been made possible by an increasing understanding of the fundamental aspects of biochemical and biophysical regulations of neuronal growth. Given its importance, several reviews cover biophysical^19–26^ and biochemical^27–30^ aspects of neuronal growth. However, most of these reviews are highly concentrated on either biochemical or biophysical aspects of neuronal growth. Given that neuroscience is increasingly becoming a highly interdisciplinary field, a review is required that covers the most fundamental aspects of neuronal growth that is comprehensible to a broader community. Such a review should be simplistic enough for researchers from interdisciplinary backgrounds to understand while also detailed enough to cover the key fundamental aspects of neuronal growth. Such a holistic review is also important because in real situations, both biophysical and biochemical cues play a role in neuronal growth and function. Besides, it has become increasingly clear in recent years that biochemical and biophysical changes are not independent of each other but, rather, are complementary. In other words, it is an action–reaction system where a biochemical action can drive a biophysical reaction and vice versa.

**FIG. 1.**
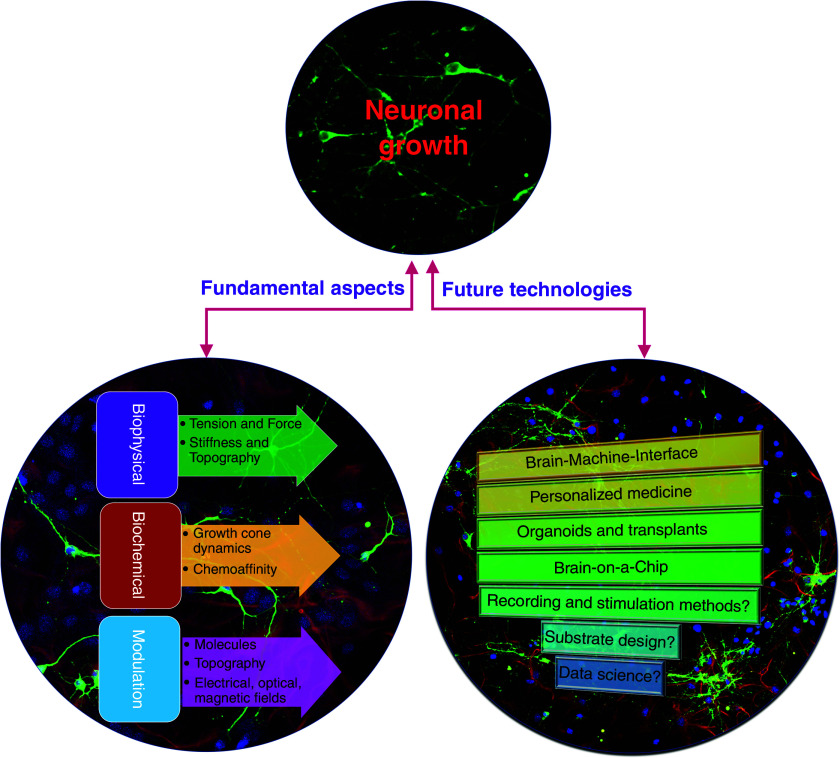
Synopsis of this review. Understanding the underlying mechanisms of neuronal growth in the brain provides an opportunity to engineer neuronal networks and develop novel future applications. This review provides an overview of the biophysical and biochemical processes that guide neuronal growth in the brain, physicochemical methods to modulate these processes, possible future applications, and some unanswered open questions in the field.

In this review, we provide a holistic view of the biochemical and biophysical mechanisms of neuronal growth by covering the fundamentals, recent progress, and unsolved questions in both these topics (Fig. 1). Throughout this review, we discuss several biochemical and biophysical mechanisms involved in neuronal growth, consciously avoiding the list of chemical reactions or long mathematical equations to make it accessible to interdisciplinary researchers from wide backgrounds. Building on the basic discussion on neuronal growth mechanisms, we discuss how these understandings are being used to modulate and control and engineer neuronal growth *in vitro*. We have also provided citations to several references to guide the readers to relevant detailed literature for each topic. Finally, we provide an outlook of future research directions in this field and outline our views on prospects of engineering and modulating neuronal growth.

## II. BIOCHEMICAL REGULATION

### A. Neuronal polarization: in vitro

In cell biology, polarity refers to the asymmetric distribution of cellular components to form structurally, morphologically, and functionally different regions in a cell. Neurons are highly polarized cells with a functionally and structurally distinct axon and dendrites attached to the soma (cell body) to facilitate the information flow between neurons. The axon and dendrites are collectively called neurites or neuronal processes. Dendrites are highly branched, relatively small processes specialized in receiving signals from surrounding cells. In contrast, an axon is significantly longer than dendrites but relatively less branched and is generally responsible for information flow out of the neuron.

**FIG. 2.**
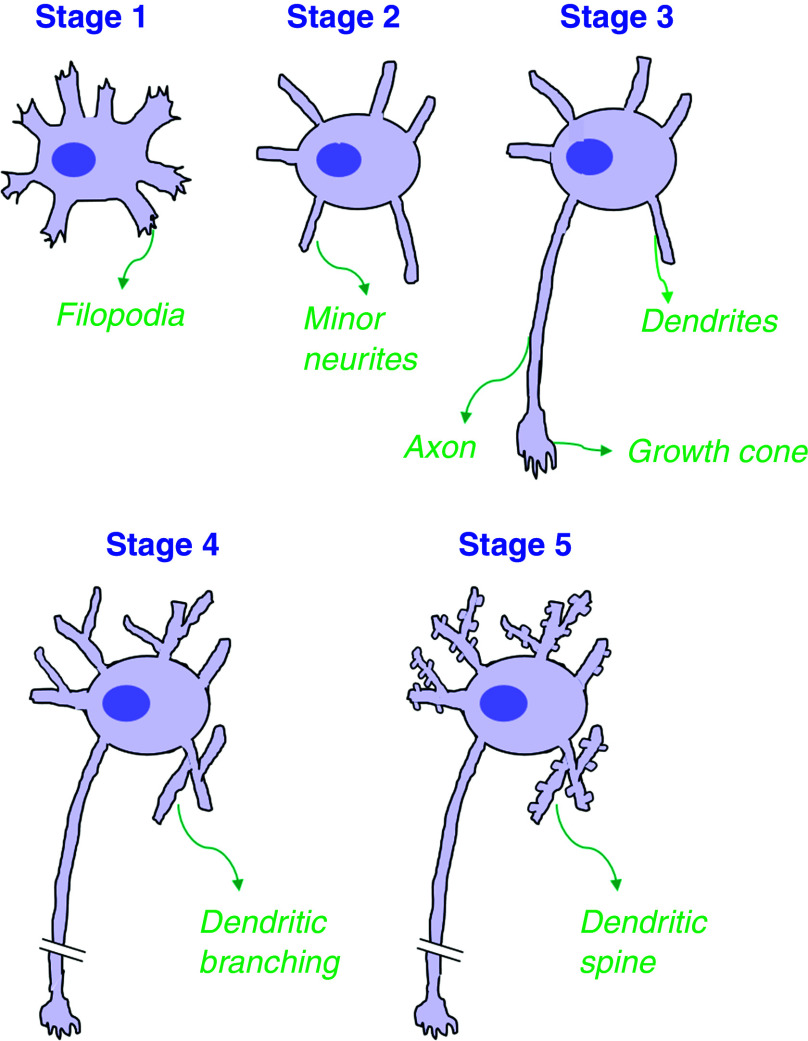
Different stages of neuronal growth in cultured hippocampal neurons (based on the work of Dotti *et al.*^32^). Stage 1: Within a few hours in culture, the newly placed spherical cells extend filopodia all around the cell body. As time progresses, the filopodia that initially extend all around the cell body break into discrete, motile patches at intervals along the cell's periphery. Stage 2: After almost half a day in the culture, the cells develop several minor processes (called neurites) that are virtually identical in morphological appearance. Once these neurites extend to a length of 10–15 *μ*m, there is little to no elongation; however, they remain motile and keep extending and retracting over small distances. Stage 3: This is the first step of neuronal polarization, where after several hours of the appearance of minor neurites, one of the several minor neurites is selected to become an axon. At this point, the chosen minor neurite grows at a significantly faster rate compared to other neurites. Stage 4: This stage is marked by significant dendritic growth. Similar to axons, dendrites also grow from minor neurites. However, considerable dendrite growth only takes place after axons have outgrown for >2–3 days. Stage 5: Subsequent maturation of dendritic and axonal arbors happens. Adapted with permission from C. G. Dotti *et al.*, J. Neurosci. **8**, 1454 (1988). Copyright 1988 Society for Neuroscience.^32^

**FIG. 3.**
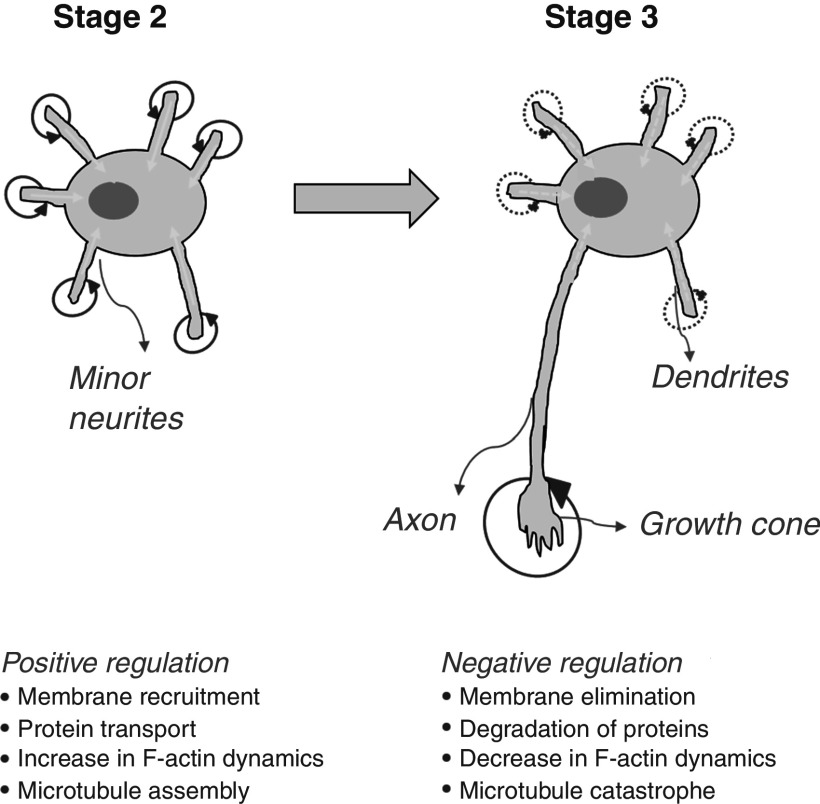
A tentative model proposed by Andersen and Bi^29^ to describe the mechanism of axon specification during neuronal growth. Initially, at stage 2, each neurite goes through continuous extension and retraction driven by a complex set of biochemical signaling cascades that form positive feedback loops (shown by red circles with arrow) and long-range negative (inhibitory) factors (shown by straight yellow arrows). A positive feedback loop at the neurite growth cone promotes neuron extension. Simultaneously, inhibitory signaling molecules, such as GTPase-activating proteins and phosphatases, counteract the positive feedback loop and cause retraction. In the transition from stage 2 to stage 3, spontaneous symmetry breaking happens, and the balance between the positive and negative feedback loop is disturbed. At this stage, one of the neurites has a powerful positive feedback loop, and this neurite develops into an axon. Concurrently, a strong positive feedback loop is counteracted through a strong inhibiting signal, which affects other neurites, and both the positive and negative feedbacks at other neurites are either reduced or wholly hindered (shown using broken lines). Increased plasma membrane recruitment; increased F-actin dynamics/microtubules, along with increased concentration; and activation of signaling molecules are some of the markers of positive cellular regulation. In contrast, negative regulation is marked by plasma membrane reduction, degradation in the local concentration of signaling molecules, decreased F-actin dynamics, and microtubule catastrophe. Adapted with permission from BioEssays **22**, 172–179 (2000). Copyright 2000 John Wiley & Sons.^29^

Banker and colleagues provided the first detailed account of morphological changes that occur during the early stages of the development of *in vitro* embryonic hippocampal neurons of fetal rats.^31,32^ They divided the early neuronal growth into five different stages, as shown schematically in Fig. 2. In stage 1, newly placed cells extend their filopodia in all directions. In stage 2, these cells develop several neurites, and each of these neurites is an equal contender for becoming an axon. Before moving to stage 3, all these neurites go through several cycles of growth and retraction (for details see Fig. 3), and in stage 3, one of these neurites starts growing much faster than the others to form an axon. During this stage, there might also be branching in the axon, and several small processes start protruding from the central axon that is connected to the soma. In stage 4, the axon and its branches elongate further, and other neurites keep going through cycles of growth and retraction before they finally form into dendrites. Eventually, in stage 5, the dendrites mature and develop the dendritic spine, and connections between different neurons are established. Here, it is important to note that even though a neuron's polarity is established in stage 3 itself, it is not until the later part of stage 4 that the complete characteristic segregation between axons and dendrites happens. It has been shown that up to early stage 4, a dendrite can develop into an axon when an already developed axon is dissected near the soma.^33,34^ The complete molecular segregation of axon and dendrites only happens at the end of stage 4 when proteins such as the glutamate receptor subunit (GluR1) and the microtubule (MT)-associated protein 2 (MAP-2) localize to the dendrites and disappear from the axon.^33,35,36^

One of the most intriguing questions in neuronal polarization is how a neuron decides which one of its neurites will develop into an axon. Based on data from studies of cell migration, axon guidance, vesicle exocytosis, and the regulation of actin and microtubule polymerization, Andersen and Bi provided a tentative molecular mechanism to describe the polarization of the axon during neuronal development.^29^ According to the hypothesis, the selection seems to be driven by a complex set of positive and negative feedback loops controlled by signaling factors (morphogens). Here, we briefly discuss the mechanism, but the molecules involved and their role in regulating the neuronal polarity are out of the scope of the current review and can be found in the literature.^37–39^

Figure 3 shows a simplified schematic of the proposed mechanism for an axon specification. Initially, at stage 2 of neuronal growth, the positive and negative feedback signals are intricately balanced and in equilibrium. However, with time, this equilibrium is disturbed by a spontaneous independent event, which may not be related to the biochemical processes happening at the neuron. This disturbance in biochemical balance pushes the system to a new, more stable equilibrium, which is the initiation of neuronal polarization in the current context. It can be understood as a case of “spontaneous symmetry breaking,” where the asymmetry emerges from symmetric, but unstable initial conditions to reach a new, more stable state (here, axon formation).^40^ First proposed by Alan Turing,^41^ the concept of spontaneous symmetry breaking is not new in biology and has readily been used to experimentally and theoretically describe the polarization in biological systems. After symmetry breaking, one strong neurite becomes the axon and has powerful positive feedback, which is counteracted with a strong negative signal, mainly influencing the other neurites where both the positive and negative feedbacks are reduced or blocked.

Although the mechanism proposed by Andersen and Bi^29^ provided very useful insight into the biochemical aspect of neuronal polarization, the mechanism is not complete. As we discuss in Sec. III, recent studies hypothesize physical forces as one of the prime modulators of neuronal polarization.

### B. Growth cone

**FIG. 4.**
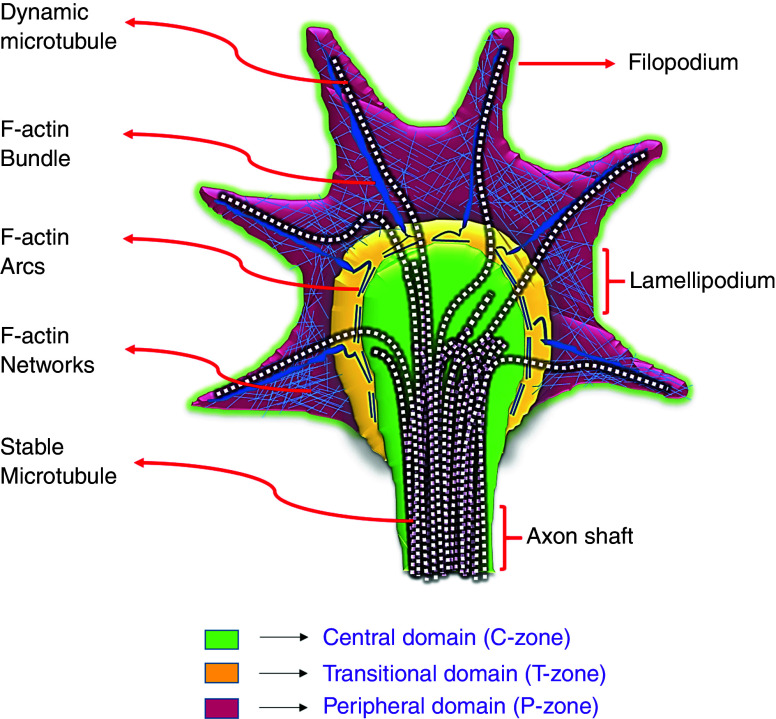
Schematic representation of cytoskeleton organization in a growth cone. The leading edge of the growth cone has highly sensitive finger-like structures called filopodia, and a sheet of membrane called lamellipodia separates each of these filopodia. Three different regions of a growth cone, based on cytoskeleton distribution, are (a) C-zone (green), (b) T-zone (yellow), and (c) P-zone (red). In an axon shaft, microtubules are organized into closely packed parallel bundles, whereas in the C-zone, they become split to extend individually through the T-zone and then into the P-zone to become aligned with the F-actin bundle (dark blue irregular lines) in the filopodium. The P-zone contains long bundles of actin filaments (F-actin bundles) and a mesh-like branched network of actin filaments (F-actin network). F-actin bundles align with microtubules to give filopodia their finger-like appearance. The T-zone sits in between the C- and P-zones. In the T-zone and the periphery of the C-zone lies the F-actin arcs (thick blue lines) composed of antiparallel bundles of F-actin and myosin II (actomyosin contractile structures). These F-actin arcs organize themselves perpendicular to F-actin bundles to form a semicircumferential ring at the tip of F-actin bundles. Reprinted with permission from E. A. Bearce *et al.* Front. Cell. Neurosci. **9**, 00241 (2015).^259^ Licensed under a Creative Commons Attribution (CC BY) license.

There are close to 100 billion neurons in a human brain, and each of these neurons makes a connection with more than a thousand target cells, and each connection (called synapses) has to be precise for the proper functioning of the brain.^42^ The question then is how can such an intricate circuit be generated with the necessary precision and reliability? Central to an axon and dendrite's ability to locate and recognize their appropriate synaptic partner is a “growth cone,” a highly dynamic and exquisitely sensitive structure situated at the extremity of growing axons and dendrites (Fig. 4).

A growth cone contains receptors that detect and bind to neurite guidance molecules to initiate intracellular signaling pathways, leading to the cytoskeleton's coordinated response, which directs the growth cone to either pause, extend, steer, or retract. A cytoskeleton is a complex system of interlinking fibers and filaments that provides the structure to a cell and helps in its locomotion and movement. Figure 4 shows a simplified schematic of the growth cone cytoskeleton. In most cases, the growth cone movement is intricately controlled by F-actin treadmilling and F-actin retrograde flow. F-actin treadmilling refers to F-actin polymerization at the leading edge of the filopodia along with F-actin severing and depolymerization at the transitional domain (T-zone) and recycling of these subunits to the leading edge.^43^ In contrast, F-actin retrograde flow refers to the centripetal, rearward movement of F-actin from the filopodial tip to the center of the growth cone. When F-actin polymerization forces are balanced with F-actin retrograde flow forces, the growth cone remains stationary. However, when a growth cone receptor binds with attractive molecular cues, tension develops, F-actin retrograde flow is attenuated, and F-actin polymerization continues to push the membrane forward (also see Sec. III A). Other than actin dynamics, MTs in the peripheral domain (P-zone) have been shown to play an essential role in steering and turning growth cones through dynein-mediated MT translocation.^44^ Further details on the mechanism of growth motility can be found in other references.^43,45–47^ Also, the growth cone's role in axonal elongation is discussed in Sec. III B 1.

While actin and MT cytoskeletons play a role in growth cone movement, many biochemical cues are involved in guiding this movement toward a target. In Sec. II C, we discuss the “chemoaffinity hypothesis,” which provides an insight into how neuronal processes find their targets using diffusible and nondiffusible molecules that act as intermediate checkpoints for navigation to their final destination.

### C. Chemoaffinity hypothesis

**FIG. 5.**
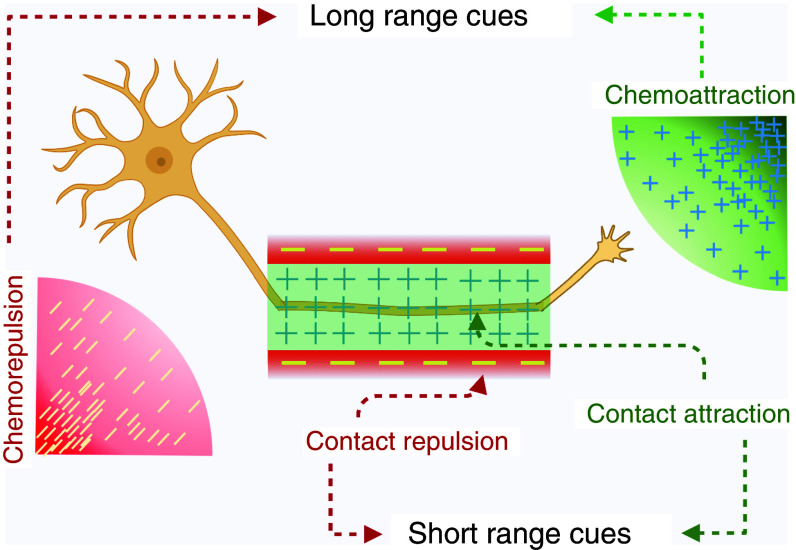
A model representation of the chemoaffinity hypothesis. There are four kinds of guidance forces that work in conjunction with each other to guide neurons to their target without getting lost. These guidance cues can be long or short range. Chemoattraction and chemorepulsion constitute the long-range guidance cues, whereas contact attraction and contact repulsion constitute the short-range guidance cues. Produced with BioRender.io.

In 1963, Roger Sperry proposed the chemoaffinity hypothesis based on his work on retinotectal projection.^28,48^ It states that neurons are guided to their target cells through a range of molecular markers that act as short-range or long-range cues for growth cone navigation. Based on the hypothesis, growth cone navigation can be broadly classified into four different mechanisms: contact attraction, chemoattraction, contact repulsion, and chemorepulsion^27^ (see Fig. 5). Contact attraction acts through adhesive molecules that are nondiffusible and are either present on the target cell surface [such as transmembrane cell adhesive molecules (CAMs)] or dispersed in/on the extracellular matrix (ECM; such as laminin and fibronectin).^27,43^ The growth cone has specific receptors that can adhere to these particular molecules and activate intracellular signaling pathways that steer the forward movement of the growth cone.^27,49,50^ Contact repulsion acts through nondiffusible surface-bound molecules (such as slits and ephrins) that do not allow sustained growth cone adherence, thereby inhibiting the growth cones' advance as they grow.^27,43,50^ The response of the growth cone to contact repulsion can range from simple deflection to axonal arrest to more dramatic changes in which the growth cone retracts and collapses.^27^ Contact attraction and repulsion are considered short-range cues for growth cones to follow to find their pathway to the target. In addition to short-range cues, there are long-range cues produced by diffusible, chemotropic molecules secreted by the axon's intermediate or final targets.^27,51^ These molecules can be either attractive (chemoattraction) or repulsive (chemorepulsion) to a growth cone based on the internal signaling pathways that are initiated in the growth cone following their interaction with specific receptors.^43,52^ Here, it is essential to note that neither short- nor long-range molecular cues work separately. Instead, they act in conjunction with each other and influence each other to initiate a complicated intracellular signaling cascade system.^52^

The prominent families of widely studied biomolecules with a well-recognized role in neuronal guidance are (a) netrins, (b) semaphorins, (c) ephrins, and (d) slits. These molecules play a role in both long-range and short-range mechanisms. Short descriptions of the role of these molecules in neuronal guidance follow.

#### 1. Netrins

Netrins can act as both chemoattractive or chemorepulsive cues based on their interaction with different types of neurons. Also, netrins can function as both long- and short-range cues. They act as long-range cues by diffusing through a source located 100 *μ*m away from the neurons.^65^ In contrast, their action as short-range cues is through immobilization on the cells.^53^ Some of the works dedicated to netrins and their receptors can be found in other references.^54–57^

#### 2. Semaphorins

The semaphorin family comprises >20 proteins that are transmembrane, secreted, or cell-substrate attached. Similar to netrins, semaphorins can also act as both long- and short-range cues. They are potent chemical repellents, and plexin receptors predominantly control their signaling.^53^ Although most semaphorins act as a repellent, some can also act as an attractant or as both attractant and repellent based on their interaction with receptors.^58^ A classic example of repulsive semaphorins is Sema3A and Sema3F, which are expressed during mouse embryonic development in tissues that surround many peripheral nerves. Through surround repulsion, they control the motor and sensory neuron projection to their normal trajectories.^58^ Reviews dedicated to semaphorins and their receptors can be found in other references.^58,59^

#### 3. Ephrins

Initially, ephrins were thought to induce a repulsive response to neurons. However, it has become increasingly clear that even ephrins can act as both attractive and repulsive cues for neurons.^60^ There are eight subfamilies of ephrins: (a) five class A ephrins that are tethered to the cell surface and (b) three class B ephrins that are transmembrane proteins.^60,61^ Ephrins bind with the receptors of the Eph family. Ephrin–Eph signaling acts exclusively as short-range cues by driving the regional differences in actin dynamics within the growth cone. Reviews dedicated to ephrins and their receptors can be found in other references.^60–62^

#### 4. Slits

Slits are large secreted proteins that act as an axon repellent through Robo family receptors. Although most slits act as short-range repellents, some of them may diffuse to a longer distance to act as a long-range axonal repellent. Reviews dedicated to slits and their receptors can be found in other references.^63–65^

#### 5. Others

Other than netrin, semaphorins, ephrins, and slits, morphogens and growth factors serve as crucial biochemical guidance cues. Among different morphogens, the most studied are the members of the Wingless-int (Wnt), and it has been shown that they can act as both chemoattractant in some cases and chemorepellent in others. Other important morphogens that take part in neuronal wiring include the transforming growth factor-*β* (TGF-*β*)/bone morphogenic protein (BMP) and sonic hedgehog (Shh) families. The role of morphogens and growth factors in neuronal wiring is an evolving field, and Henríquez and Osses compiled a list of important papers related to the topic.^66^

As discussed, these guidance molecules are of prime importance for controlling neuronal growth. Keeping that in mind, in Sec. IV A, we discuss different methods through which these guidance cues can be controlled *in vitro* for neuronal growth.

## III. BIOPHYSICAL REGULATION

Although most of the earlier studies on neuronal growth were only concentrated on biochemical regulations, many recent works point toward a substantial involvement of biophysical cues in deciding and maintaining the fate of a neuron both *in vivo* and *in vitro*. Moreover, it is becoming increasingly clear that the responses of a neuron to biochemical or biophysical cues are not independent of each other but, rather, are complementary. Biochemical regulations can be understood as machinery to induce the biophysical response or vice versa. In this section, we discuss some of these biophysical mechanisms involved in neuronal growth and their correlation with already-discussed biochemical regulations.

### A. Substrate stiffness and topography

It is becoming evident that mechanosensing of the physical environment followed by the initiation of mechanotransduction (discussed in Secs. III A 1–III A 3) pathways is strongly involved in regulating neural cell development and its functioning.^19^ One of the most common means for biophysical modulation of neuronal growth is changing the substrate's stiffness and topography on which the cells grow. It has been shown that both surface stiffness and micro- and nanoscale topographies of the substrate influence the behavior of *in vitro* neuronal network formation and activity.^67–72^ For example, in a recent study, Koser *et al.* reported that local stiffness gradient guides the *Xenopus* retinal ganglion cell (RGC) neuron growth both *in vitro* and *in vivo* and that changing the brain tissue stiffness leads to axonal pathfinding errors.^70^ Our group has also shown that by growing neuronal cells on nanopillar arrays, both neuronal growth and activity can be efficiently modulated.^73^ Given its importance in neuronal growth, in the Secs. III A 1–III A 3, we discuss the fundamental mechanism by which nanotopography and stiffness influence neuronal growth. We first provide a general description of cell–microenvironment interactions, which may also apply to neurons, and at the end of this section, we discuss the neuron-specific mechanotransduction studies.

#### 1. Mechanotransduction pathways in stiffness sensing

**FIG. 6.**
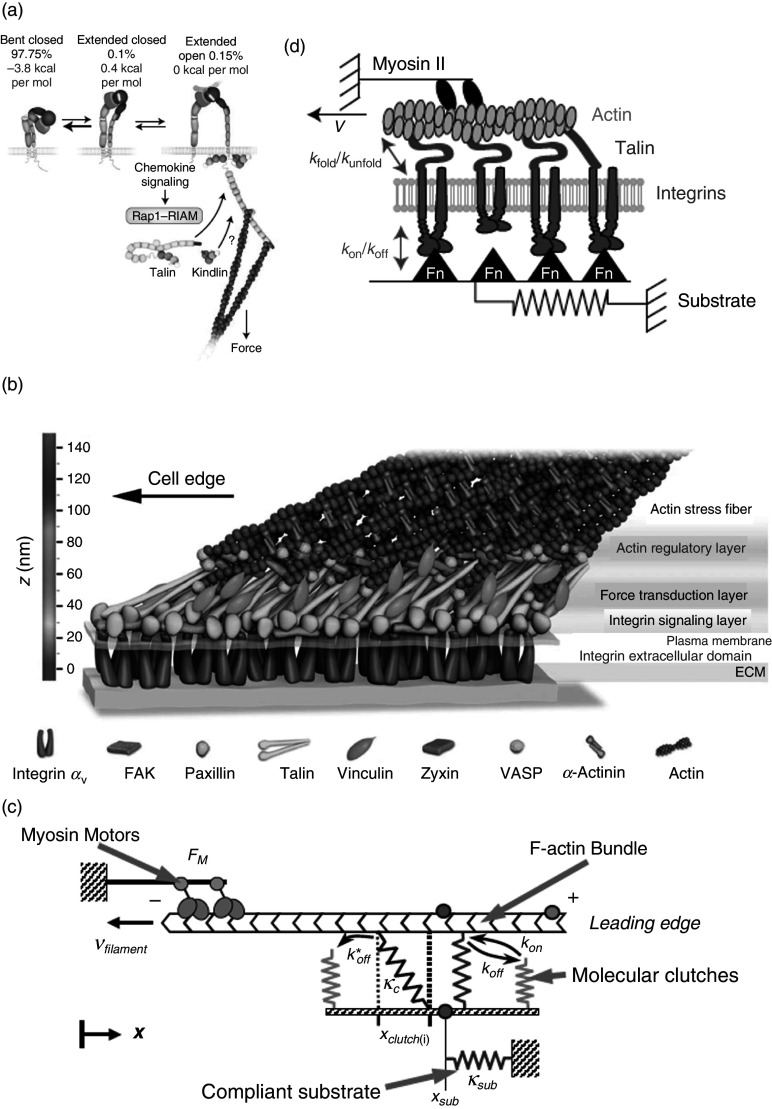
(a) Integrin can exist in three different configurations, including bent closed, extended closed, and extended open. Thermodynamically bent closed configuration is the most stable, and integrins predominantly occupy this configuration, but it can also fluctuate between extended closed and extended open configurations under thermodynamic equilibrium.^74^ The right end of the figure shows that the binding of integrin with ligand and adaptors applies a force from the actin cytoskeleton to the adaptors; at the same time, a resistive force acts at the point of integrin and substrate binding. Reprinted with permission from Z. Sun *et al.*, Nat. Cell Biol. **21**, 25–31 (2019). Copyright 2019 Springer Nature.^76^ (b) A model showing the nanoscale localization and molecular architecture of proteins involved in focal adhesion. Starting from the bottom, the separation between inner plasma membrane and substrate was measured ∼32 nm, whereas actin was separated from the plasma membrane by another ∼40 nm. In between the plasma membrane and actin layer are the integrin-mediated signaling proteins [focal adhesion kinase (FAK), paxillin], cytoskeletal adaptors (vinculin, talin, zyxin), and actin-regulatory proteins [vasodilator-stimulated phosphoprotein (VASP), α-actinin]. Overall, the architecture shows three spatial and functional compartments of focal adhesion: an integrin signaling layer, a force transduction layer, and an actin regulatory layer. Reprinted with permission from P. Kanchanawong *et al.*, Nature **468**, 580–584 (2010). Copyright 2010 Springer Nature.^79^ (c) A physical model to describe how substrate stiffness influences the motor clutch mechanism of substrate–cell binding. It shows a myosin motor driving the retrograde flow to exert a continuous force *F_M_* (toward left) at a velocity *ν_filament_*. The rate constant *k_on_* and *k_off_* are, respectively, the rates at which the molecular clutch can engage or disengage with F-actin bundles to counter the rearward retrograde flow. The clutches' successful engagement with the actin bundles builds tension with a spring constant *κ_c_*, and the clutches are stretched to strain *χ_clutch(i)_*. The total tension built across all the engaged springs amounts to a pulling force that is counteracted by the deformation in substrate with a tension equivalent to spring constant *κ_sub_*. As it becomes evident, the mechanical resistance to loading is determined by *κ*_c_ and *κ*_sub_. Reprinted with permission from C. E. Chan and D. J. Odde, Science **322**, 1687 (2008). Copyright 2008 American Association for the Advancement of Science.^82^ (d) Figurative representation of modified molecular clutch model proposed by Elosegui-Artola *et al.*^84^ The model assumes that the actin filament is pulled by myosin motor at a speed of *v*, and they are connected to a compliant substrate through integrin and its adaptors, such as talin. The model also includes force-mediated unfolding of talin (i.e., above a certain force threshold, talin unbinds to strengthen the cell–substrate binding), which is not accounted for in motor clutch model shown in (c). The talin folding/unfolding rates are represented by *k_fold_* and *k_unfold_*, respectively, and *k_on_* and *k_off_*, respectively, represent the binding and unbinding rate of integrin and its adaptor clutches with the substrate. The substrate deformation is modeled using a single elastic spring with variable stiffness. Adapted with permission from A. Elosequi-Artola *et al.*, Nat. Cell Biol. **18**, 540–548 (2016). Copyright 2016 Nature Publishing Group.^84^

Integrin has long been recognized as a prime mediator of molecular–mechanical linkage between a substrate and a cell.^75^ Integrin-mediated mechanotransduction plays a critical role in the regulation of several aspects of cellular physiology, including cell proliferation, viability, differentiation, and migration.^76,77^ Integrins are composed of a large ectodomain (the part of a membrane protein that extends out of the cell), which mediates substrate binding, a transmembrane domain, and a short cytoplasmic tail that indirectly binds with the actomyosin (actin–myosin complex) cytoskeleton through integrin adhesomes [Fig. 6(a)]. They can exist in three different conformations, including bent closed, extended closed, and extended open configurations, each having different ligand-binding capabilities^76^ [Fig. 6(a)]. A shift from bent/extended closed to an extended open configuration is termed “integrin activation.”^76^ The prevailing view on mechanosensitive integrin activation is that it only requires the binding of adaptor proteins (such as talin and kindlin) to the tail end of the integrin [Fig. 6(a)]. However, most recently, Li and Springer^74^ and Li *et al.*^78^ performed a detailed thermodynamic study to show that an ultrasensitive integrin activation requires both adaptor binding and cytoskeletal force.

Kanchanawong *et al.* used super-resolution light microscopy to show that the molecular architecture of integrin-mediated focal adhesion consists of at least three domains, including an integrin signaling layer, a force transduction layer, and an actin regulatory layer^79^ [Fig. 6(b)]. They also found that the vertical arrangement of these different focal adhesion components was highly consistent across different shapes and sizes of focal adhesion and was not at all correlated with the area or aspect ratio of focal adhesion.

Moreover, to explain integrin-based mechanosensing a “motor clutch” mechanism has been proposed. Mitchison and Kirschner first proposed this concept to describe the mechanism of force exertion on a substrate by actin retrograde flow to initiate a forward movement in the neuron.^80^ The molecular clutch refers to the mechanical linkage that forms between the substrate and F-actin through cell adhesion molecules, which in response, create a friction slippage bonding to transmit traction force that reduces the F-actin retrograde flow and advances the leading edge.^81^ Chan and Odde extended this model to test its response to substrate stiffness.^82^ They constructed a simple stochastic physical model that treated molecular clutches and the substrate as simple Hookean springs [Fig. 6(c)], and they experimentally substantiated their model against embryonic chick forebrain neurons transferred on substrates with different stiffness. They found that on a compliant substrate, there are two distinct regimes of traction force dynamics: (a) “frictional slippage” regime on stiff substrates and (b) “load-and-fail” regime on flexible substrates. On a stiff substrate, the clutches engage quickly to form a tension exceeding their breaking strength, leading to shortened F-actin/clutch interaction time. A shortened F-actin/clutch engagement duration does not provide enough time for other clutches to engage and strengthen the force; therefore, on a stiff substrate, sufficient traction force cannot be generated. On the other hand, on a soft, compliant substrate, the rate at which the tension is built on the clutch is reduced, which increases the time for F-actin/clutch engagement, and more clutches can now engage in increasing the total tension to achieve sufficient traction force. As a result of traction force, the retrograde flow of the myosin motor is reduced, and a forward movement of the growth cone is accomplished. In due course, the load on one of the clutches exceeds that breaking strength, leading to the clutch's detachment followed by a rapid failure and disengagement of all other clutches, causing the cell to rest back to its initial position.^82^

Chan and Odde's model has been verified in several other experiments, but it does not explain the observation of systems where there is a monotonic increase in force transmission with stiffness.^82^ Most recently, Elosegui-Artola *et al.* improved on the Chan and Odde's model and included the molecular behavior of integrin, talin, and vinculin into the mathematical model to provide a more accurate prediction of integrin-based mechanosensing and force transduction for a wide range of substrates [Fig. 6(d)].^83,84^

#### 2. Mechanotransduction pathways in topography sensing

**FIG. 7.**
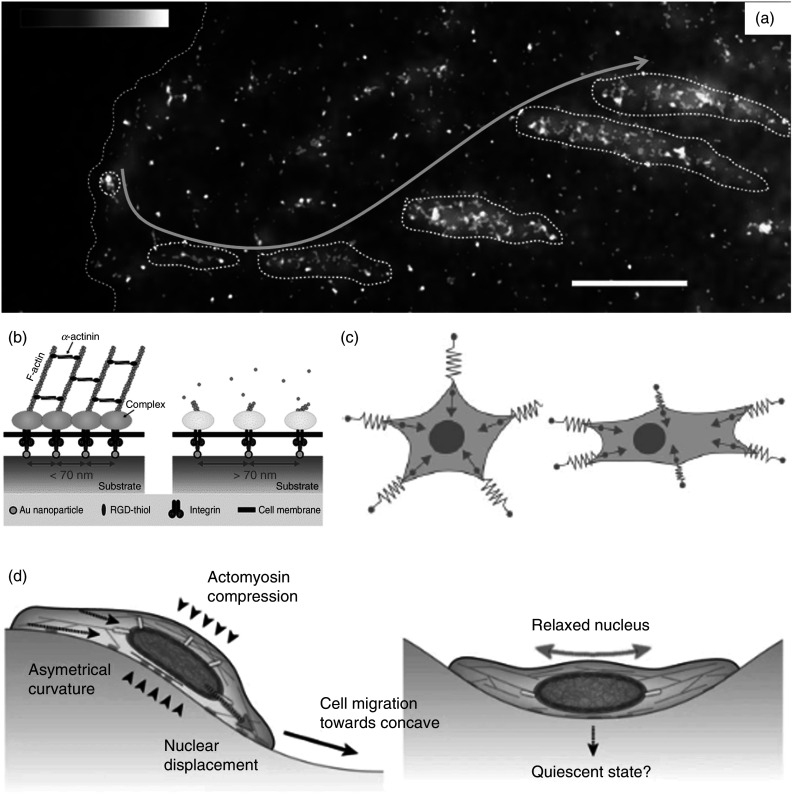
(a) Different levels of adhesion in a mouse embryonic fibroblast spread on the fibronectin-coated substrate for 30 min. The encircled dotted lines (follow the yellow arrow) from left to right indicate the nascent adhesions, maturing adhesions, and mature adhesions, respectively. Reprinted with permission from R. Changede and M. Sheetz, BioEssays **39**, e201600123 (2017). Copyright 2016 Wiley Periodicals.^89^ (b) A schematic representation showing that an interspacing of >70 nm between two subsequent binding sites significantly limits integrin clustering and reduces cell functions. Reprinted with permission from M. Arnold *et al.*, ChemPhysChem **5**, 383–388 (2004). Copyright 2004 Wiley-VCH.^90^ (c) A schematic representation of Bischofs and Schwarz's model. It assumes that each point of cell contact with its environment can be assumed as a linear spring. Furthermore, depending on the isotropic or anisotropic environment, cells can adopt round or stellate morphology or a polarized morphology, respectively. Reprinted with permission from I. B. Bischofs and U. S. Schwartz, Proc. Natl. Acad. Sci. U.S.A. **100**, 9274–9279 (2003). Copyright 2003 The National Academy of Sciences.^94^ (d) Schematic representation of “curvotaxis” modeled by Pieuchot *et al.*^96^ It shows that the cell migrates to concave valleys to achieve nuclear relaxation. Reprinted with permission from L Pieuchot *et al.*, Nat. Commun. **9**, 3995 (2018). Licensed under a Creative Commons Attribution (CC BY) license.^96^

Other than stiffness, the topography of the 2D substrate or 3D scaffold can also regulate the neuronal outgrowth, axonal guidance, and neuronal activity. A complete understanding of how a cell senses its topography is not very well understood. However, there is sufficient evidence to support that cells can detect their environment's nanotopographical information down to <10 nm using extremely fine nanoscale membrane projections.^85–87^ As discussed in Sec. III A 1, the interaction of cells with the extracellular environment is primarily mediated by integrin binding and integrin clustering, which leads to an intracellular signaling cascade and regulates the engagement of the cellular cytoskeleton in the adhesion process. Just after its interaction with the substrate, the cell forms a force-independent nascent adhesion of size of a few tens of nanometers.^88,89^ Arnold *et al.* reported that a universal-length scale for integrin clustering and activation ranges between 58 and 73 nm.^90^ This result has been supported by other authors, and it has been shown that if the global average spacing between cell adhesive sites is >70 nm, the cell functions reduce significantly due to restricted integrin clustering^91^ [Fig. 7(b)]. Depending on the force and other extracellular factors (such as stiffness and topography), the nascent adhesion can disassemble quickly within 1–2 min or mature to focal adhesion. The size of maturing or matured focal adhesion can be anywhere from a few hundred nanometers to several micrometers^92^ [Fig. 7(a)]. Because the nascent/focal adhesion sizes lie in micro- and nanometer ranges, it is imperative that the growth and behavior of cells can be modulated through the topographical patterns of micro- or nano-sizes.^93^ Hence, nano- and micropatterns can be used for modulating cellular adhesion, which in turn modulates the cellular behavior. Section IV C provides a more detailed account of how nanotopography is being used to modulate neuronal growth.

Bischofs and Schwarz proposed a mathematical model to show how a cell might organize in 2D/3D soft media due to active mechanosensing.^94^ They assumed that a cell would form several contacts with its environment, and each of these will sense a different kind of mechanical encounter with its environment^94^ [Fig. 7(c)]. They assumed that the point–contact interactions with the local elastic environments are similar to that of a linear spring, so for each point contact, there can be the same or different *K*, depending on whether the cell is in the anisotropic or isotropic environment, respectively. In other words, the cell acquires a round or star-like morphology for cell spreading on micro-/nano-sized patterns arranged isotropically because the *K*s are equal at all points of the cell–matrix interaction. In contrast, in an anisotropic medium, the force generated in one direction will be larger than other directions, and the cell will orient itself, preferably in that direction. Such directionality in cell spreading has been seen in substrates with oval micropillars and nanogratings and substrates with stiffness gradients.^95^ Their analysis also showed that in cases where cells cannot deform the substrate, it will only respond to topographical cues, whereas if it can deform the substrate, it responds to both the stiffness and topographical cues.^95^ In another biophysical model, Pieuchot *et al.* showed that a cell responds to cell-scale curvature variations and will migrate to its closest curvature minimum while avoiding the convex regions.^96^ They proposed that the cell migrates to concave valleys to achieve nuclear relaxation, and this migration is guided by the interplay of an actomyosin compressive cortex and a stiff nucleus and not by the ECM–cell contact–triggered signaling [Fig. 7(d)].

The mechanotransduction mechanisms discussed in Secs. III A 1 and III A 2 are not specific to neuronal cells but, rather, are a general description of the cell's interaction with its surroundings through topographical or stiffness cues. In Sec. III A 3, we discuss some of the mechanotransduction studies specific to neurons and their role in deciding the fate of neurons.

#### 3. Neuron-specific mechanotransduction studies

Neurons extend throughout the body, crossing through tissues that consist of complex multilayer environments displaying a broad spectrum of morphologies, stiffness, topography, and size scales. Therefore, given its importance, the effect of topography and stiffness on different kinds of neurons has been widely reviewed by several authors.^95,97–103^ Therefore, without going into detail here, we discuss only a few of the studies where researchers have done a detailed analysis to show how neurons might interact with the extracellular environment to influence their morphology and behavior.

Koch *et al.* studied the effect of substrate stiffness on different kinds of neurons, comparing the neurite growth of rat dorsal root ganglion (DRG) and hippocampal neurite on substrates with varying stiffness. They showed that while the growth of hippocampal neurons was not dependent on substrate stiffness, the DRG neurons had maximum outgrowth when the substrate had a Young's modulus of ∼1000 Pa.^104^ They also found that the peripheral nervous system (PNS) neurons showed a higher traction force compared to central nervous system (CNS) neurons. Similarly, Koser *et al.* reported that the local stiffness gradient guides the *Xenopus* RGC neuron growth both *in vitro* and *in vivo*, and changing the brain tissue stiffness leads to axonal pathfinding errors.^70^ These results clearly show that the sensitivity of neurons to substrate stiffness is neuron specific and not universal.

**FIG. 8.**
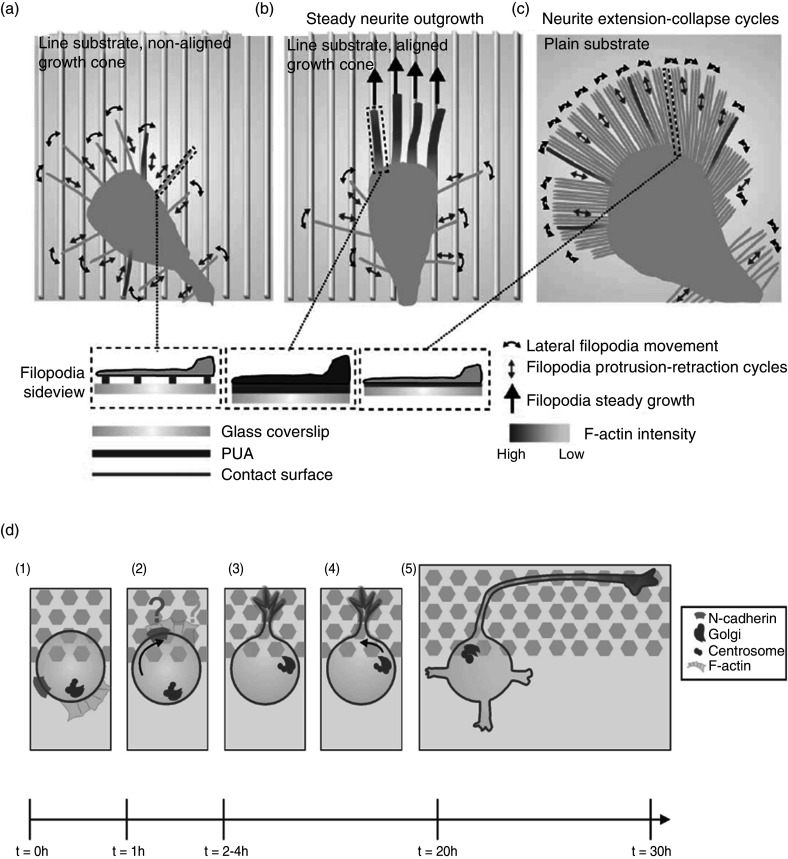
(a) Filopodia scans the line substrate and goes through protrusion and retraction cycles. Also, each filopodium senses the different adhesion levels to the ECM, and only a few can align on the line substrate. (b) After several protrusion and retraction cycles, multiple filopodia become aligned with the lines and stabilize through the F-actin cytoskeleton's recruitment. However, even at this point, not all filopodia are aligned, and several unaligned, unstable filopodia keep operating on the distal part of the growth cone. (c) On the planar substrate with a uniform contact surface, all filopodia extend simultaneously and equally, and none of them has enough chance to become stabilized by the F-actin cytoskeleton, leading to an increased number of collapses and retraction of the growth cone. PUA, polyurethane-acrylate. Reprinted with permission from K.-J. Jang *et al.*, PLoS One **5**, e15966 (2011). Licensed under a Creative Commons Attribution (CC BY) license.^105^ (d) Events showing neuronal polarization on a patterned pillar substrate. Initially, when a neuron is at the interface of the planar and pillar array (stage 1), a patch of N-cadherin accumulates toward the pillar (stage 2), and F-actin aligns itself toward the nanotopographical feature. Next forms the first sprout of neurite from the cell (stage 3), and the Golgi and centrosome are inducted at the planar/pillar interface (stage 4). Finally, from this point of first growth, the neuron polarizes. Reprinted with permission from L. Micholt *et al.*, PLoS One **8**, e66170 (2013). Licensed under a Creative Commons Attribution (CC BY) license.^106^

Jang *et al.* compared the neuron orientation and outgrowth on planar substrate and substrates with lines of nanoscale sizes.^105^ They found that line substrate with nanoscale features was sufficient to orient the neuronal outgrowth and that there was a robust increase in neuronal outgrowth on line substrates compared to planar substrates. Through a detailed analysis, they proposed that growth cone filopodia play the most crucial role in the sensing of nanoscale features [Figs. 8(a)–8(c)]. In another report, Micholt *et al.* proposed that neuron polarization under topographical stimuli happens in five different stages^106^ [Fig. 8(d)]. Although they do not discuss the role of tension in polarization, the polarization may be due to directional tension (discussed in Sec. III B 3) induced due to forces exerted through N-cadherin contacts with substrate.

Furthermore, to understand how neuronal differentiation is influenced by nanotopography, Schulte *et al.* compared the neuronal differentiation of PC12 cells on nanostructured ZrO_2_ substrates with that of poly-L-lysine (PLL)–coated glass and flat zirconia.^107^ They found that neuronal differentiation was higher on nanostructured ZrO_2_ substrates compared to both PLL-coated glass and flat zirconia with or without the nerve growth factor. They performed ultrastructural characterization of the cell/nanostructure interface and detailed proteomic profiling of nanostructure-induced neuritogenesis and mechanotransduction of PC12 cells on nanostructured zirconia (ZrO_2_) substrates and planar substrates. Based on the results obtained, they postulated that higher differentiation of neurons on nanostructured ZrO_2_ substrates compared to the planar substrate resulted from graded focal adhesion formation due to limited integrin clustering, which in turn activates mechanotransduction pathways that modify the activation dynamics of transcriptional factors susceptible to mechanosensitive inputs. These studies show that neuronal fate can largely be decided by altering the surrounding environment's topography and stiffness. Another biophysical cue that plays a major role in neuronal growth and its behavior is force. In Sec. III B, we discuss the role of force in the modulation and control of neuronal growth.

### B. Force and tension

Many processes during neuronal growth involve motion, which means that universal laws of force must govern all those processes. The role of some sort of mechanical tension in axonal growth was evident as early as the 1930s.^108^ Experimental evidence at that time suggested that once neurons form synaptic connections during early embryonic development, their growth rate increases significantly in coordination with expanding surrounding tissues.^109^ For example, at a peak growth rate of the human fetus, a motor axon grows at a rate of ∼50 *μ*m/h, i.e., almost 100 times faster than the growth rate of an axon before synapse formation. This rapid extension of neurons after synapse formation was termed as “passive stretching” by Harrison^108^ or “towing” by Weiss.^110^ Bray provided the first experimental evidence of axonal elongation induced by externally applied mechanical tension in 1984.^109^ Moreover, in 2002, Lamoureux *et al.* were able to show that any minor neurite can be decided to become an axon through the application of an appropriate tension at its tip, even in the presence of an already developed axon.^111^ In Sec. III B 1, we discuss the role of force in axonal elongation and polarization.

#### 1. Force and axonal elongation

**FIG. 9.**
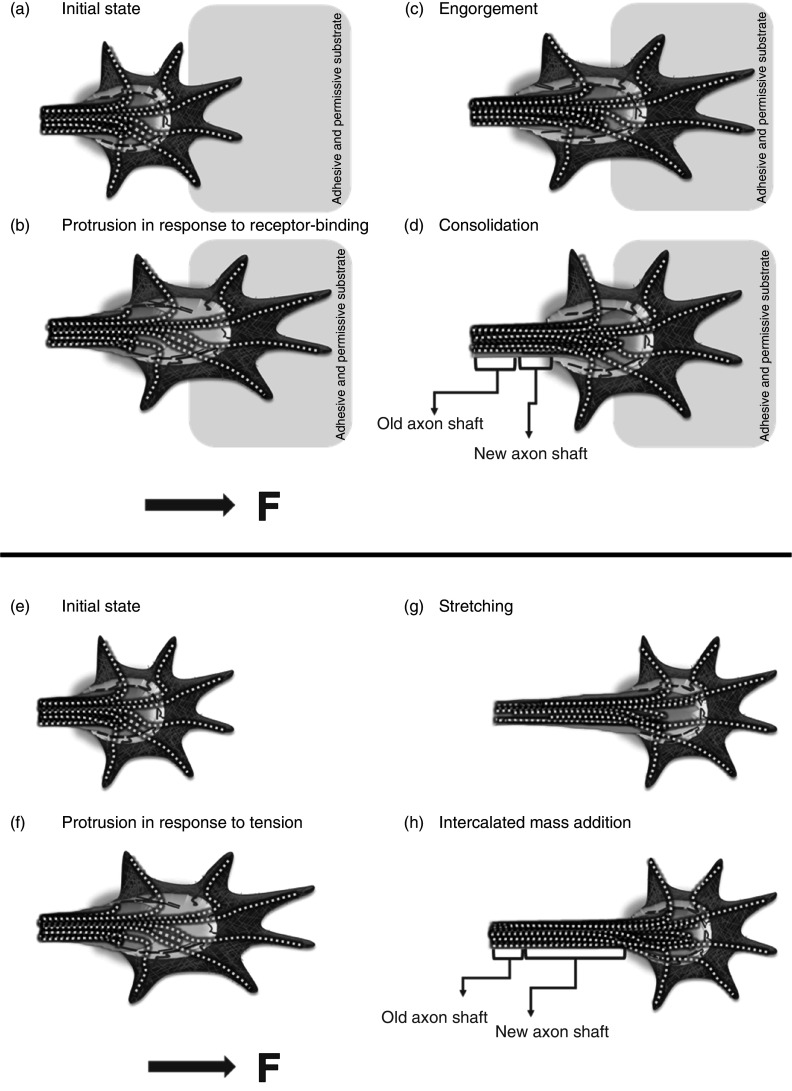
(a)–(d) The model for growth cone–mediated axonal elongation. Axonal elongation in response to the force exerted due to binding of the growth cone receptor with a permissive and adhesive substrate (a) happens in three different stages: protrusion, engorgement, and consolidation. (b) At the protrusion stage, the binding of the distal portion of the growth cone receptor with a permissive substrate strengthens significantly, and the retrograde flow of F-actin firmament is attenuated (creating space for microtubule advance), while F-actin polymerization continues in front, leading to a forward movement of filopodia and lamellipodia and elongation of the P- and T-domains of the growth cone. (c) Engorgement occurs when microtubules invade the protrusion, causing fast transport of cell organelles and vesicles (mitochondria, endoplasmic reticulum) to the tip of the C-domain of the growth cone. At this stage, the C-domain moves forward to align below the P- and T- domains. (d) Finally, the C-domain's consolidation happens through mass addition at the proximal end of the growth cone to form a new segment of the axon shaft. (e)–(h) The model for axonal elongation in the presence of mechanical tension. Similar to the previous model, the application of a mechanical tension leads to the protrusion (f) in the growth cone and a forward extension of filopodia due to attenuated retrograde flow and a continuous F-actin polymerization. However, unlike engorgement, this model assumes that the axon goes through viscoelastic stretching and thinning (g), pulling together the axon's distal region and the C-domain forward. Finally, over a few hours, the stretched portion of neurons recovers its original volume through intercalated mass addition (h). Also, stretching and intercalation seem to be cyclic processes such that one can precede or supersede the other or happen simultaneously. Arrow in figures denote the direction of force. Based on Fig. 2 in Suter and Miller.^22^

The mechanism of axonal elongation has been a long-debated subject and is still evolving. The conventional growth cone–mediated axonal elongation model assumes that the axonal outgrowth is mediated only by the growth cone and happens in three steps, namely protrusion, engorgement, and consolidation [Figs. 9(a)–9(d)]. First proposed by Goldberg and Burmeister in 1986, this model has been at the center stage of research involving axonal elongation.^112^ The second model is the stretch and intercalation model [Figs. 9(e)–9(h)] of axonal growth, which is relatively new and is particularly useful to describe the axonal growth in the presence of mechanical tension. These two models differ in ways that the mass addition happens during axonal growth. The first model assumes that mass addition occurs at the growth cone through organelles' engorgement (vesicles, mitochondria, etc.) and assembly of microtubules in the central domain (C-zone) of the growth cone. In contrast, the former implies that mass acquisition occurs along the length of the neuron through the assembly and deposition of new materials. Several reviews have covered growth cone–mediated axonal outgrowth,^30,113–116^ whereas other works provide details on the model of tension-induced elongation.^117–120^ A more recent model has been proposed by Miller and Suter that assumes a combination of subcellular forces and subcellular fluidic motion.^26^

Although these models can describe axonal growth in most cases, there are still cases where these models do not fit. Given that both the models assume that the mass addition requires transport of organelles and cellular components to the distal region of the axon, it is logical to think that the maximum rate of elongation of a neuron can only be equal to the rate of most slowly transported essential components (e.g., tubulin that has a transport rate of ∼1 mm/day).^22^ However, there have been reports of neuronal growth rate as high 8 mm/day.^121^ Furthermore, a large animal can undergo enormous neuronal growth during its development phase even when integrated axons do not have growth cones from which they can extend. One example of the most extreme axonal growth is the spinal cord axon growth rate in blue whales, which can be as high as 3 cm/day.^122^ To describe such extreme neuronal growth under stretch, most recently, Purohit and Smith presented a mathematical model, but they do mention that a more detailed experimental investigation is required to reach any conclusion.^123–125^

Given that the mathematical models remain the most accepted tool to describe force-induced neuronal growth, in Sec. III B 2, we discuss two of the most accepted models of force-induced neuronal growth, namely Dennerll's and O'Toole's models.

#### 2. Mathematical models for axonal elongation under tension

**FIG. 10.**
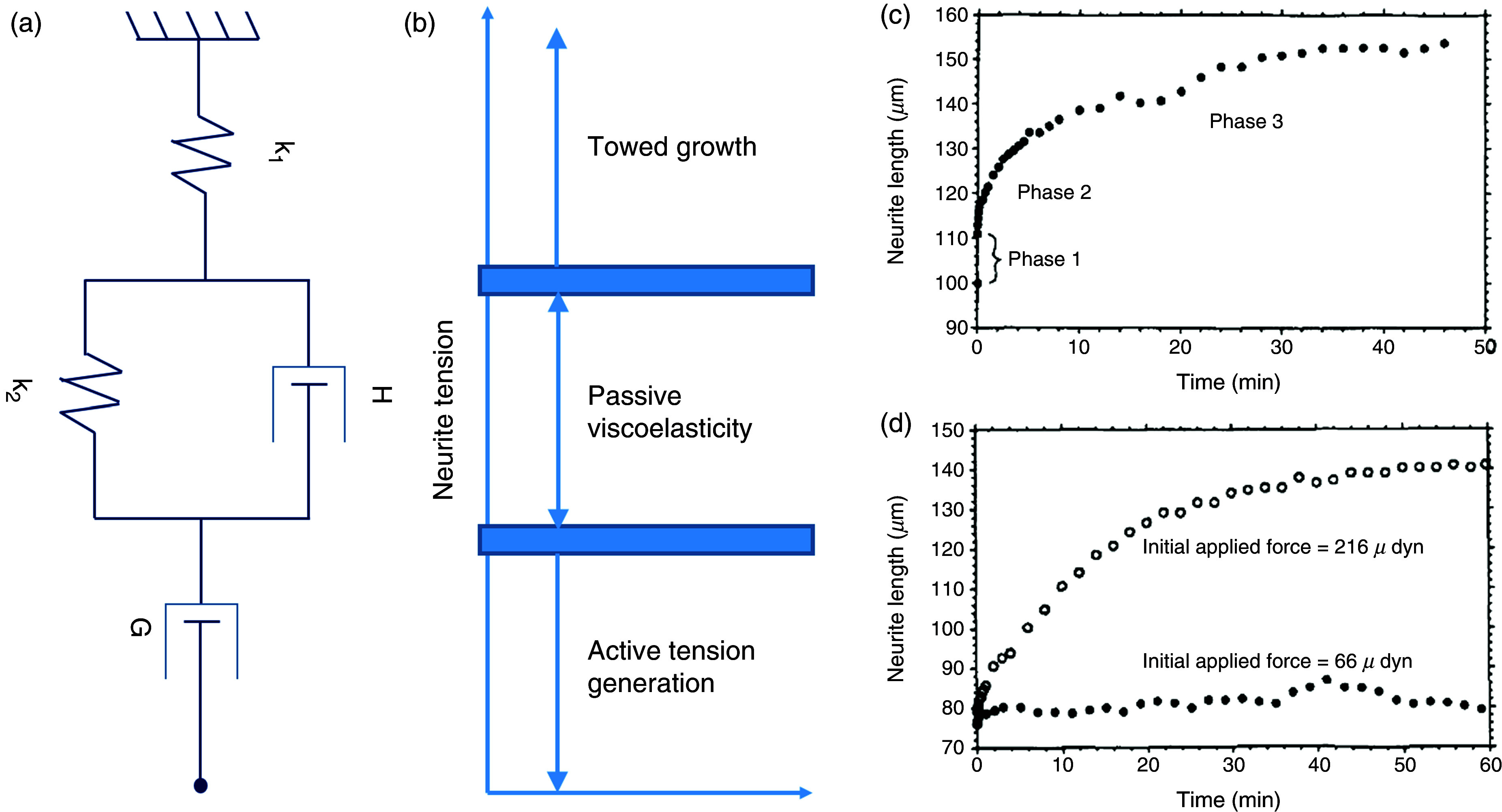
(a) A three-element model proposed by Dennerll *et al.*^125^ The model assumes a relatively stiff spring constant of k_1_, which accounts for solid deformation under tension; a Voight element, which accounts for passive viscoelasticity causing damping and reduced tension; and an additional dashpot in series, which accounts for neuronal elongation leading to tension dissipation such that there is a linear relationship between applied tension and elongation rate. (b) A figurative representation of three possibilities of tension-regulated axonal response. It shows that above some given tension set point, the axons are stimulated to elongation, whereas below a given tension threshold, neurons are stimulated to retract, and between these tension thresholds, neurons act passively as a viscoelastic solid. (c) Three phases of neuronal response under tension. Phase 1 is a rapid increase in neurite length under tension. Phase 2 slows damping and saturation in neuron length. Phase 3 continues elongation over a long duration. (d) If the tension is not sufficient, neurons only go through phase 1 and phase 2 and do not show long-duration elongation (i.e., phase 3). However, with a tension higher than a few nanonewtons, the neurons showed continuous elongation for a longer duration. Reprinted with permission from T. J. Dennerll *et al.*, J. Cell Biol. **109**, 3073–3083 (1989). Copyright 1989 Rockefeller University Press.^125^

Dennerll *et al.* provided the first biophysical model to mathematically describe the axonal elongation and retraction in response to an externally applied mechanical tension.^125^ They assumed that the deformation response of a neurite under tension has both fluid- and solid-like properties [Figs. 10(a) and 10(b)]. One of Dennerll's model's most critical aspects was that it was able to explain an experimentally observed three-step process in the axonal response to an applied mechanical tension. More specifically, the model predicted two phases of neuronal responses in a low-force regime (approximately <1 nN) and three phases of neuronal responses in a high-force regime. In a low-force regime, the two phases included a rapid stretching for a short duration followed by longer viscoelastic damping. However, if the force (approximately >1 nN) was sufficient to induce axonal elongation, the neuron had three phases of response to tension. Phase 1 and 2 were similar to the former, but the neuron applied with high tension had an additional phase that consisted of a continued elongation for a longer time [Figs. 10(c) and 10(d)]. In other words, the model was also able to show that to induce neuronal growth, a sufficiently high tension was required, which was consistent with experimental results.

**FIG. 11.**
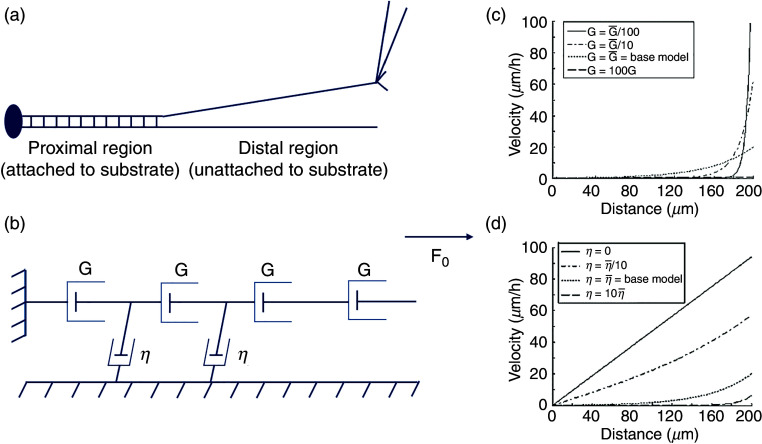
(a) Diagrammatic representation of a neuron under towing. The distal portion of the neuron adjacent to the towing needle is detached from the substrate, even though the neuron itself remains firmly attached to the substrate through several adhesion sites in the proximal region. (b) O'Toole's model assumes each segment of an axon as the free or frictional Burgers element. This model treats the distal part of the neuron, which is under constant tension, as a series of free dashpots because under continual force, the Burgers element's behavior is dominated by its free dashpots, whereas the proximal region of neuron adhering to the substrate is treated as a friction dashpot with the constant of friction given by *η*. (c) The rate of axonal elongation is directly correlated to the axonal viscosity. When the viscosity is very high, there is almost no elongation in the neuron (broken line lying straight near the x axis), whereas for low viscosity, the elongation rate increases rapidly and may lead to rupturing of the neuron. (d) Similarly, for a large value of *η* (i.e., strong adhesion), forces dissipate quickly, and very little bulk transport is observed. In contrast, when there is no adhesion (*η* = 0), force is not dissipated, and the velocity profile of bulk transport is linear. Reprinted with permission from M. O'Toole *et al.*, Biophys. J. **94**, 2610–2620 (2008). Copyright 2008 The Biophysical Society.^120^

O'Toole *et al.* extended Dennerll's model [Figs. 11(a) and 11(b)], assuming that the axon is a series of Burgers elements.^120^ This allowed them to study the effect of tensile forces at each point along the length of an axon. Furthermore, by including the effect of the adhesion in the neuron's proximal regions, they could account for both the tension generation at the growth cone (i.e., distal part of the neuron) and its dissipation along the length. They postulated that the two factors that play the biggest role in defining the velocity profile of an axon under tension include (a) axonal axial viscosity, given by g, and (b) the constant of friction, given by *η* (accounts for the interaction between the substrate and the axon).^120^ Their model predicted that the neurons' velocity profile is linear in the region not attached to the substrate and nonlinear in the neuron segment attached to the substrate. Moreover, the elongation rate of the substrate-attached segment of the axon was directly proportional to F_0_/(G*η*)^1/2^, where F_0_ is the constant tension along the distal region of the axon, and G is a product of the g and axon cross-sectional area A [Figs. 11(c) and 11(d)]. One of the most critical aspects of the model is that it predicted whether a neuron will follow growth cone–mediated growth or stretch growth (Fig. 9), depending on the force at the tip of the growth cone and the adhesion between the substrate and the neuron, which is consistent with the previous report.^126^ More specifically, the model showed that if the traction force due to axon adhesion on a substrate reduces rapidly while moving away from the growth cone and decreases to zero along the axon, the neuron follows growth cone–mediated elongation, whereas when the traction force reduces gradually toward the length of the axon, it supports stretch growth. Another critical aspect of their model was that it predicted that the axon thinned dramatically due to the transport of a sizeable cytoskeletal mass away from the cell body when neurons elongated rapidly in a towed experiment. In contrast, no such thinning was observed in a naturally growing axon.

#### 3. Axon polarization using force

In addition to enhancing the growth rate of neurons, tension can also help the specification or polarization of an axon. Here, we discuss some of the studies where force and tension alone could decide which neurite becomes an axon. Initially, it was thought that neuronal polarization requires one of the neurites to achieve a critical length, after which the said neurite starts elongating at a faster rate than other neurites. However, now it is becoming increasingly clear that tension plays a vital role in deciding the fate of a neuron and plays a central role in the axonal specification.^39,126–129^ This is not surprising considering that neurons are in constant subjugation of tension during their development.^130,131^ Lamoureux *et al.* provided the first direct evidence that any neurite can be stimulated to become an axon solely by application of mechanical tension.^111^ In another set of experiments, Gomez *et al.* simultaneously compared the effect of nerve growth factor (chemical cues) and microtopography (which indirectly influenced the tension across neurites) on neuronal polarization and found that the impact of microtopography was more pronounced compared to immobilized nerve growth factors.^132,133^ In yet another report, Micholt *et al.* showed that only surface topography was sufficient to determine the neuronal polarization *in vitro*.^106^

A clear understanding of the mechanism of how tension plays a role in the axonal specification is still elusive. One of the possible mechanisms can be that the immature neurites go through several cycles of retraction and growth before one of the neurites becomes stabilized through cell–cell interaction or cell–substrate interaction to create enough tension to initiate polarization.^38,39,111^ However, some of the research findings have also shown that the dynamics and transport of microtubules in the axon are directly affected by tension and may play some role in tension-induced axonal specification.^126,134^

So far, we have covered several different biophysical and biochemical mechanisms of neuronal growth. One of the prime motivations of understanding these mechanisms has been to achieve a high level of artificial control over neuronal growth. In Sec. IV, we discuss some of examples of how an increased understanding of neuronal growth mechanisms is helping researchers to control *in vitro* neuronal growth.

## IV. NEURONAL GROWTH CONTROL USING EXTERNAL CUES

An increased understanding of different biophysical and biochemical cues of neuronal growth has led researchers to develop innovative methods to control neuron growth *in vitro*. These methods for controlling neuronal growth can have a far-reaching impact in the biomedical field and are critical for fulfilling the human ambitions of brain–machine interface and brain-on-a-chip. Therefore, in this section, we cover some of the basic neuronal growth mechanisms that have been used for controlling neuronal growth *in vitro*.

### A. Molecular guidance cues

The most basic requirement for neuron survival *in vitro* is that the substrate's surface (or 2D/3D extracellular matrix) should support the cell attachment.^135^ However, this is not always the case, and most often, commonly used substrates, such as glass, do not promote cell adhesion. In such cases, to achieve cell growth compatibility, a surface is usually coated or functionalized with (a) polymers (such as polyamino acids and polycations like polylysine) that can interact with the negatively charged cell membrane, (b) ECM components (such as laminin, collagen, and fibronectin), (c) self-assembled monolayers (SAMs), (d) cell adhesion proteins, and (e) hydrophobic modification using biological molecules such as polysaccharides.^135–140^ The process of surface patterning may also allow for the formation of biphasic regions of adhesive and nonadhesive surfaces by selectively coating the regions with cell-compatible biomolecules.

**FIG. 12.**
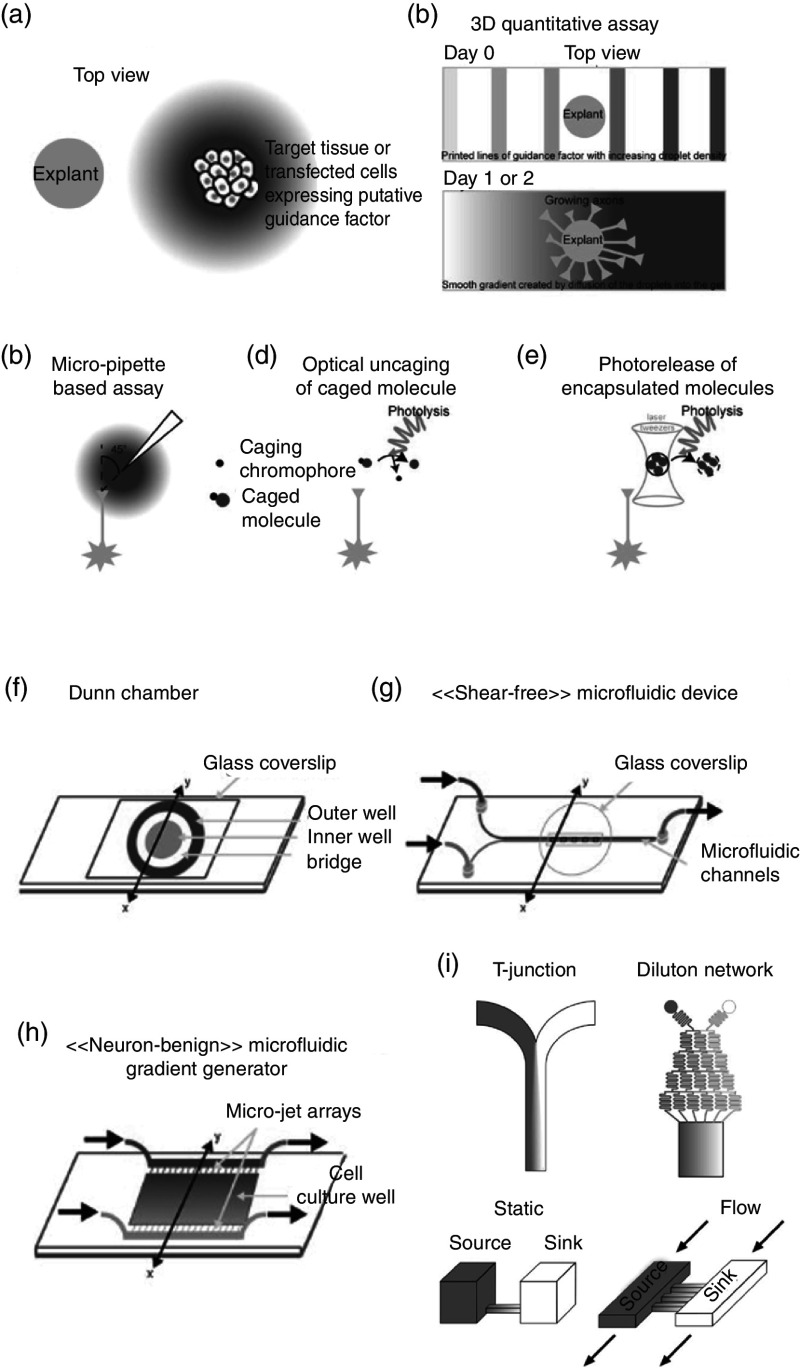
(a) In a classical coculture, a tissue explant containing the nascent neurons is cultured with a target tissue in a semisolid biologically derived matrix, such as collagen gel or plasma clot. The target tissue expresses guidance factors, which influence the growth of explant neurons. (b) In modern and improved quantitative co-culture, guidance molecules are patterned on top of a 3D matrix containing the explant. The stripped molecules diffuse through the 3D matrix to create a predictable gradient. (c) In a micropipette assay method for neuron guidance control, guidance molecules are introduced to the culture through a pipette (generally held at a 45^o^ angle) with a known volume of guidance molecules in the vicinity of the growth cone. (d) Optically induced uncaging can be used to release guidance molecules with higher spatiotemporal control of neuron guidance. (e) Photorelease is similar to uncaging in concept, but the guidance molecule's release is achieved through photolysis (i.e., breakage of a bond using light). (f) A schematic representation of a Dunn chamber. The chamber has an inner well (gray circular area), an outer well (red circular area), and an annular area (white circular area) where diffusion takes place. Here, neurons can be exposed to an almost linear gradient of guidance cues. (g) A shear-free microfluidic device has a Y-shaped fluidic microcircuit that is interfaced with a semipermeable membrane. The cells are cultured in the microwell, having a gradient of guidance cues. (h) A simplified schematic of a neuron-benign microfluidic device. It has a central open-surface reservoir connected and fed laterally through two microchannels termed “sink channel” and “source channel.” Neurons are cultured into the reservoir, having the gradient of the guidance molecules. Reprinted with permission from I. Dupin *et al.*, J. Neurosci. **33**, 17647 (2013). Licensed under a Creative Commons Attribution (CC BY) license.^142^ (i) Different kinds of microfluidic channels along with an approximate diffusion profile created by those channels. Reprinted with permission from B. Lin and A. Levchenko, Front. Bioeng. Biotechnol. **3**, 39 (2015). Licensed under a Creative Commons Attribution (CC BY) license.^161^

However, an *in vivo* neuron's molecular environment is far more complex, and only cellular adhesion is not sufficient. The neural connection, as discussed in Sec. II C, is guided by chemotropic molecules. Therefore, to achieve precise control on the guidance of neurons *in vitro* requires precise control of molecular guidance cues as well. There have been several different ways of controlling neuronal growth *in vitro* by controlling guidance molecules.^141^ As a rule of thumb, an ideal guidance method should be to achieve the following goals: (a) precise control of molecular cue gradients both in space and time, (b) easy compatibility with neuronal culture methods and with live-cell imaging, and (c) responsiveness to large throughput acquisition of data.^142^ We briefly describe some of the *in vitro* methods (Fig. 12) for controlling spatiotemporal distribution of neuronal guidance molecules.

#### 1. Co-culture guidance assay

Co-culture refers to a cell culture technique where two or more cell types are grown together such that one affects the other. In an initial version of 3D coculture assay, an explant containing nascent neuronal cells was grown together with a target tissue in a biologically derived 3D matrix. The target tissue expressed the guidance molecules for explant neurons, which controlled the neuronal growth. One of the significant advantages of this technique is that it allows for direct visualization of results, and several different experiments can be conducted at the same time. In contrast, one of the major drawbacks is that it does not enable quantitative control of guidance cues. Several different kinds of 3D quantitative co-culture assays have been developed in recent years to achieve more precise, reproducible, and stable gradients of molecular cues.^143–145^ Dedicated reviews on the co-culture techniques can be found in other references.^146–148^

#### 2. Methods for local control of guidance cues

One of the easiest ways to control the local concentration of guidance cues is through a micropipette. In this technique, a micropipette containing a known concentration of guidance cues is held at an angle for the steady release of the guidance cues in the vicinity of the growth cone. Often, micropipettes can also be coupled with a microfluidic platform for more advanced spatiotemporal control.^149^ Moreover, a more advanced technology that mimics the guidance control similar to the micropipette is microfluidic multi-injector (MMI).^150^ MMI can generate arbitrary overlapping gradients of multiple guidance cues through simultaneous exploitation of microfluidic integration and the actuation of many pulsatile injectors and has a very high level of temporal controllability.^151^

An alternative approach to achieving spatial and temporal precision of local guidance cues is through using light. This method requires a light-sensitive caged compound that can encapsulate guidance molecules and can liberate them under light stimulation through uncaging or photolysis.^152–155^ Given that light can be both modulated and controlled in time and amplitude, light-induced uncaging can be used to produce a rapid and repetitive release of biomolecules or can be finely tuned for graded changes in the magnitude of molecular release.^153^

#### 3. Dunn chamber for diffusible control of guidance cues

A Dunn chemotaxis chamber allows the study of cell behavior under a linear concentration gradient of guidance molecules. When combined with fluorescent cell labeling techniques and time-lapse microscopy, the Dunn chamber can allow for real-time analysis and visualization.^156^ The Dunn chamber is made from glass. It contains two circular wells separated by an ∼1-mm-wide annular platform set at 20 *μ*m below the chamber surface^157^ [Fig. 12(f)]. For chemotaxis study, cells adhering to a coverslip are inverted and lowered onto the chamber such that there is a small slit left in the outer well to transfer the molecules. After molecules have been transferred to the outer well, it diffuses radially to the inner well while the cells remain positioned over the annular bridge (because it is 20 *μ*m below the chamber surface).^158^ Since the first report in 1991, Dunn chemotaxis has become a popular tool for guidance studies mainly because of its simplicity and possibility of real-time visualization of the response of multiple neurons at the same time.^142^

#### 4. Microfluidics for diffusible control of guidance cues

Microfluidics refers to the science of manipulating and controlling small quantities of fluids generally ranging from microliters (10^−6^) to picoliters (10^−12^) through networks of channels of dimensions tens to hundreds of micrometers. In recent times, with microtechnology advancements, microfluidic devices have become a popular choice for *in vitro* neuronal culture. One of the most critical aspects of these microfluidic devices is that they can be designed to selectively target individual processes of neurons independent of each other in a highly controllable and reproducible manner, which was not possible with traditional methods.^159,160^ There are several different designs of microfluidic devices, some of which are shown in Figs. 12(g)–12(i). Moreover, given the importance of microfluidics and its application of neuronal culture, it has been widely covered in several of the referenced reviews, and readers should refer to them for more details.^141,161–167^

#### 5. Micropatterning for control of substrate-bound guidance cues

Another common technique to control guidance cues in *in vitro* neuronal culture is micropatterning. Micropatterns are formed by selectively coating the substrate with different forms of micrometer-scale architectures of guidance cues.^168,169^ Several different kinds of micro-architectures can be formed to achieve either continuous or gradient profiles of guidance molecules. It is probably one of the most efficient and fastest methods for controlling the distribution of guidance molecules on 2D substrates.^170–174^

To conclude this section on control of molecular guidance cues for neuronal growth, Table I lists the common advantages and limitations of methods used to control guidance cues during *in vitro* neuronal growth and references for further information. In addition to controlling neuronal growth biochemically, neuronal growth can be controlled by controlling the neuron's biophysical aspects. Table II summarizes some of the different ways in which biophysical aspects of neuronal growth can be modulated to control *in vitro* neuronal growth.

**Table I.** Advantages and limitations of methods used for controlling molecular guidance cues to modulate *in vitro* neuronal growth.

Method	Advantages	Limitations	References
Co-culture guidance assay	A variety of cell types can be cultured together. It also allows for direct and real-time visualization of results.	Conventional co-culture methods do not allow for quantitative control of guidance cues.	146–148
3D quantitative co-culture assay	Same as co-culture assays but with more quantitative control over guidance cues.	The relative change in concentration of guidance cues is relatively small along the growth cone's width. The method requires relatively expensive equipment.	146–148
Micropipette injection	Extremely localized control of guidance cues is possible.	Low throughput: At a time, only one cell can be exposed to a varying concentration of guidance cues.	150, 151
		Poor control due to the high possibility of human error.	
Dunn chemotaxis chamber	Low-cost systems available commercially. Can be combined with optical methods for real-time tracking and observation.	The system is limited to dissociated cells. The gradient shape is almost linear and cannot be modified.	142, 156–158, 175
Light-induced photolysis or release	High spatiotemporal control of guidance cues.	Finding the appropriate compound is difficult.	152–155
Micropatterning and microprinting	Precise characterization of neuronal response to graded guidance cues.	Require high-cost instruments and lack temporal control.	^170–174^
Microfluidic devices	High spatiotemporal control with high throughput.	Limited gas and nutrient exchange due to constrained environment.	141, 161–167
		In some cases, there might be sheer stress that may damage cells.	

**Table II.** Different methods for biophysically modulating *in vitro* neuronal growth.

Method	Hypothesis and mechanisms involved	References
Tension	Force can be used to enhance neuronal growth rate without compromising its function.	109, 111, 121–123, 176, 177
Magnetothermal	Localized heat generation due to rotating magnetic nanoparticles can modulate neuronal growth.	178, 179
Magnetoelectric	Magnetoelectric effect can be used to remotely control voltage-gated channels	180
Magnetic force	Magnetic force can be used to orient magnetic nanostructures to one specific direction to achieve guided force-induced neuronal growth.	181–184
Topography	Topography can be used to modulate neuronal growth in several ways by influencing cell spread, adhesion, and activity.	19, 98, 103, 185–190
Electric field	Growth cone direction can be controlled by the application of a voltage, most likely through the redistribution of charged proteins.	191–199
Optics	Light may influence neuronal growth by (a) optically enhanced actin polymerization and (b) localized heat-induced microtubule and actin polymerization.	196–200

### B. Tension

**FIG. 13.**
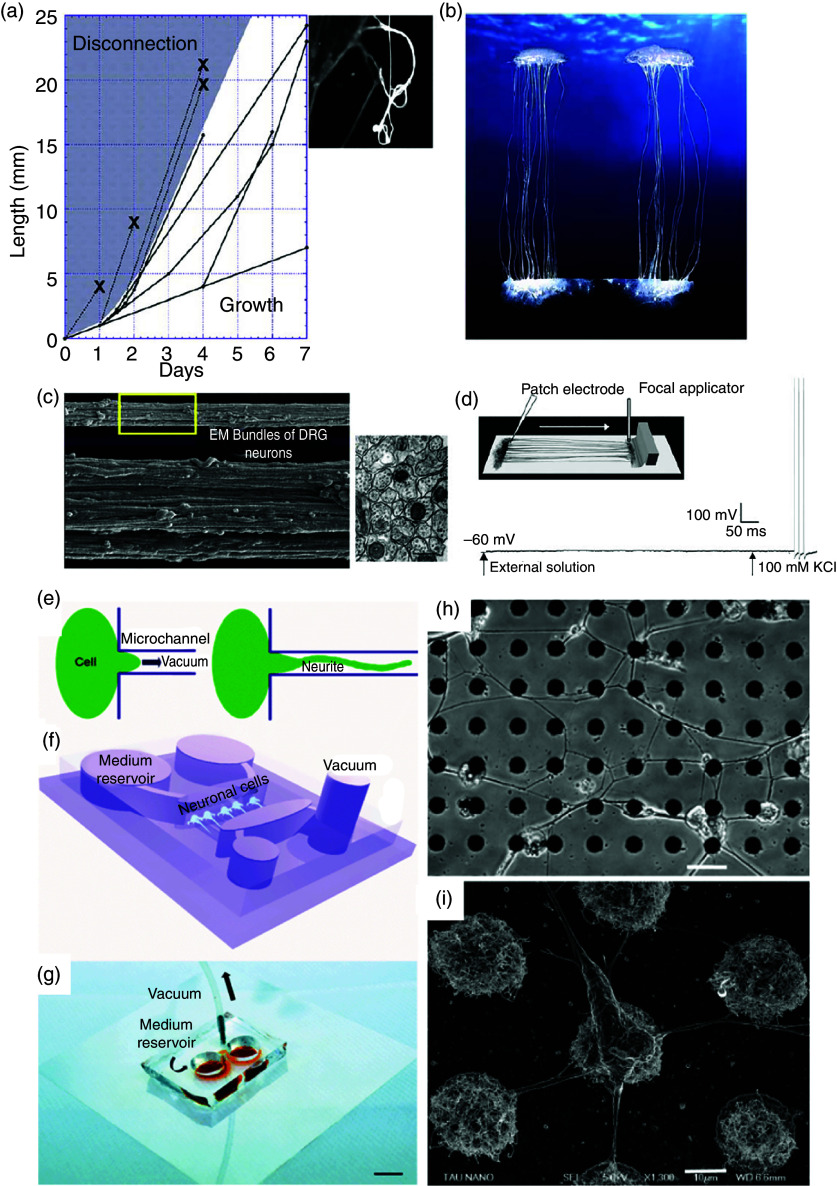
(a) The condition within which stretch-induced neuronal growth happens. The conditions should be maintained similar to the white region; otherwise, neurons disconnect. (b) A photograph of a dorsal root ganglion that was extended from 100 *μ*m (adjacent length across) up to 5 cm in <2 weeks of stretch. (c) Ultrastructure of neurons showing that they were healthy. The extended neurons were found to be of greater diameters compared to nonstretched axons taken from a sister culture. This might occur due to the natural adaptive process of development, which facilitates structural integrity. (d) Schematic of a setup used to check the responsiveness of stretched neurons. The graph below shows the potential transmission across neurons when one of the ends was exposed to KCl. Reprinted with permission from D. H. Smith, Prog. Neurobiol. **89,** 231–239 (2009). Copyright 2009 Elsevier.^122^ (e) Comparative schematic representation of neuronal growth under vacuum applied to the microfluidic device. (f) The neuron is cultured in the central portion of the microfluidic well, which is connected through other wells through a microchannel for feeding culture medium and application of vacuum. (g) A photograph of the microfluidic device connected to a vacuum pump through a pipe. The scale bar is 1 cm. Reprinted with permission from T. D. Nguyen *et al.*, Lab on a Chip **13**, 3735–3740 (2013). Copyright 2009 Royal Society of Chemistry.^203^ (h) and (i) The carbon nanotube microanchors on which neurons are cultured. Neurons attached to these anchors act as though they are under constant tension. The scale bars are 50 μm in (h) and 10 μm in (i). Reprinted with permission from S. Anava *et al.*, Biophys. J. **96**, 1661–1670 (2009). Copyright 2009 Biophysical Society.^208^

Initial experiments on tension-induced control of neuronal growth were performed using towing needles.^109,111,176^ However, most probably, one of the significant milestones in tension-induced neuronal growth has been extreme stretch growth of integrated axon tracts using a specially designed experimental setup first reported by Smith *et al.* in 2001.^177^ Since then, the same group has published several papers on the subject,^121–123,177^ and have been able to demonstrate that this method can be exploited to engineer transplantable living nervous tissue constructs. Figures 13(a)–13(d) shows some of the selected results on extreme stretch growth along axon tracts from Smith's lab. Similar results through another process have been reported by Abe *et al.*^200^ They induced indirect nerve elongation through the leg-lengthening process at a 1 mm/day rate, with only a small percentage of neurons showing degradation after 70 mm of growth. Most recently, Sahar and colleagues have proposed devices that can mimic this axon stretch growth *in vivo*.^201,202^

In another report, Nguyen *et al.* demonstrated another way to control *in vitro* neuronal growth using tension in a microfluidic device.^203^ Figures 13(e) and 13(f) show the working model of the device, and Fig. 13(g) shows a photograph of the setup. To apply tension, they connected one of the microfluidic wells that are connected to cultured cells (through microchannels) to a pump through a tube. They achieved a growth rate of 12.5 *μ*m/h under an applied pressure of −400 Pa in PC12 neurons. Furthermore, they calculated that for given dimensions of microchannel openings and an ∼3 *μ*m neurite diameter, the applied pressure of −400 Pa created an initiating force of ∼20 000 pN and an extending force of ∼2800 pN. Given that the method can be integrated with microfluidic devices, it can be highly useful because microfluidic devices are already prevalent for neuronal culture.

At the same time, more precise control of neuronal tension on a single neuron level can be achieved by using a micropipette attached to a suction pump and a positively charged polymer bead. This method relies on the connection formed between the axon and microbeads coated with a positively charged polymer [poly-D-lysine (PDL) and netrin], which pulls the axons upward.^204–206^ This method can further be combined with microfluidic devices to achieve a higher level of control and reproducibility.^205,206^ Moreover, Magdesian and colleagues have shown that this method can be used to achieve a growth rate >0.33 *μ*m/s over millimeter-scale distances, which is several times higher than the normal *in vitro*–cultured neuron growth rate.^205,206^

### C. Topography

Other methods that have been used for neuronal growth control and modulation is through micro- and nanoscale patterning and topography. The micropatterning method relies on the premise that neurons will have a different level of adhesion on different geometries or materials. Therefore, these adhesion-induced tensions can be exploited to control neuronal growth. One way to apply tension using micropatterning is by culturing neurons on isolated anchoring sites. While the neuron cell body attaches to one of these anchors, the axon remains under constant tension due to pulling from other anchors. Hanein *et al.*^207^ and Anava *et al.*^208^ have shown that carbon nanotubule (CNT)–based anchors [Figs. 13(h) and 13(i)] can very well mimic the mechanical tension to influence neuronal networking.

**FIG. 14.**
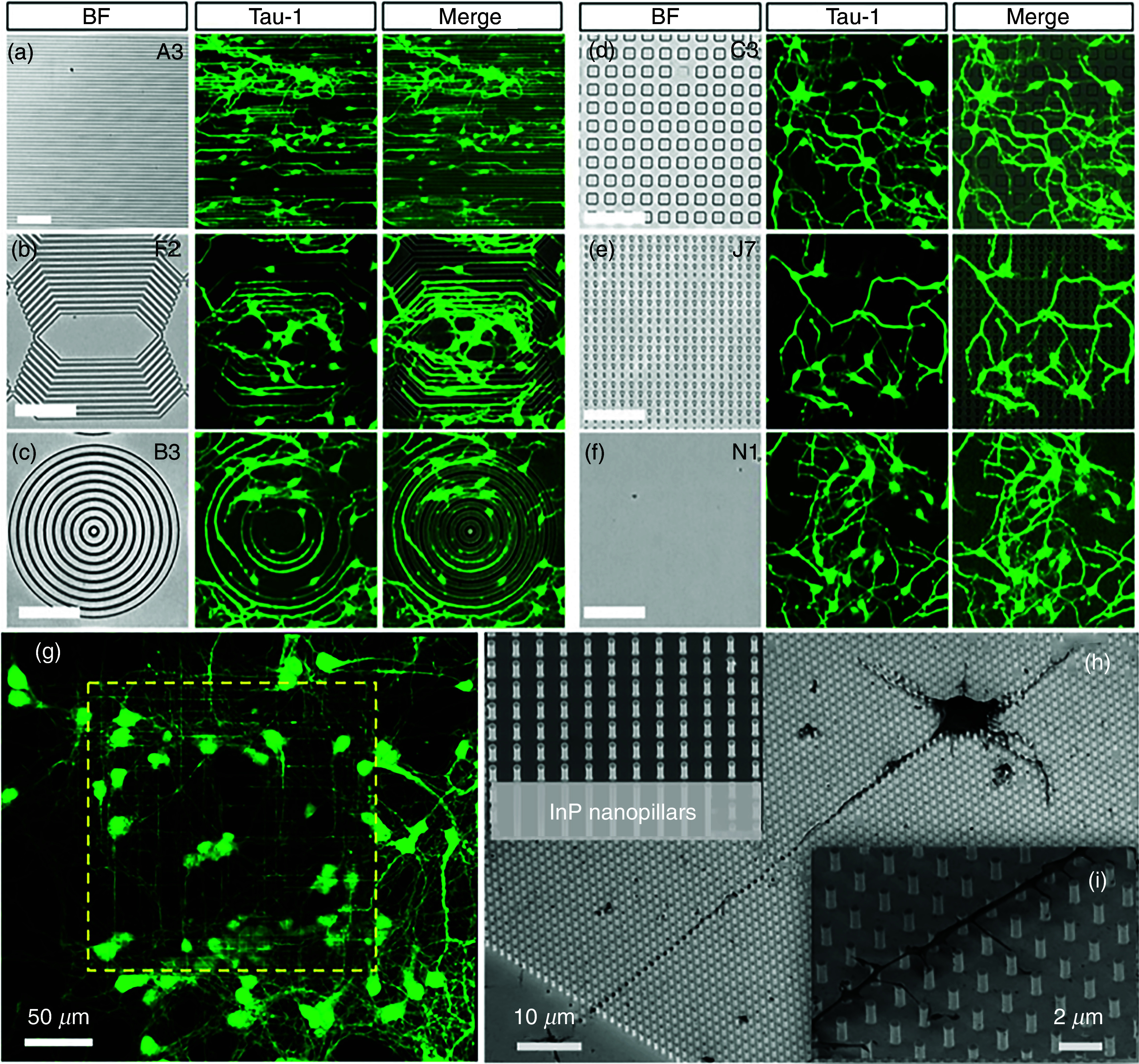
(a)–(e) Axonal outgrowth on different 2D microscale topographical patterns after 2 days *in vitro*. It is apparent in (a)–(c) that the axons follow the transition edges between ridges and grooves for guidance, whereas on isolated and sporadic patterns, such as the one shown in (d) and (e), neuronal growth had minimal guidance effect. (f) A planar control structure with no microtopography and almost no neuronal guidance. (a)–(f) BF stands for bright field, scale bar: 100 μm. Reprinted with permission from W. Li *et al.*, Sci. Rep. **5**, 8644 (2015). Copyright 2009 Biophysical Society.^210^ (g) Rat hippocampal neurons labeled with *β*-III-tubulin grown on a vertically aligned InP nanowire array show neurite alignment on the nanowires. The dashed square highlights the area with the nanowires. (h) Scanning electron microscope images showing vertically aligned InP nanowires and a mouse cortical neuron growing on them. (i) Fine details of the neurites originating from the neuron in (h). Reprinted with permission from V. Gautam *et al.*, Nano Lett. **17**, 3369–3375 (2017). Copyright 2017 American Chemical Society.^73^

Besides, researchers have also widely used different kinds of topographies to control neuronal adhesion and tension, which in turn can control neuronal polarization,^106,128,132^ growth rate and neuronal regeneration,^136,187,209^ neuronal guidance,^73,210^ cell morphology and alignment,^211,212^ and branching^107,210^ in *in vitro* cultures. Several different kinds of topographical variations, such as anisotropic topography (e.g., alternating grooves and ridges, gratings, and parallel fibers), isotropic topography (e.g., pillars, posts, cones, and linear-, circular-, and dot-shaped patterns), and random topographical gradient (micro- and nano-roughness) have been used by researchers to influence neuronal growth. To perform a detailed and systematic study of the influence of topography on neuronal growth, Li *et al.* fabricated a range of 71 different isotropic and anisotropic nano/micropatterns on a single chip.^210^ A few of the different micropatterns they used are shown in Figs. 14(a)–14(f). As expected, their results showed that topography plays a significant role in the determination of axon and dendrite length as well as their branching and guidance.^210^ Similarly, we have also demonstrated that neuronal growth can be engineered using nanoscale topography in the form of nanopillars.^73^ Figures 14(g)–14(i) show the neurite growth of rodent neurons on InP nanowires. Our work showed for the first time that neurite growth could be guided along the nanoscale topography of vertically aligned InP nanowires. We further showed that when presented with such topographical cues, neurons form a highly interconnected network with each other and exhibit synchronized calcium activity, implying intercellular communications via synaptic connections.^73^

Moreover, recent advances have made it possible to generate several-meters-long microfibers with tunable morphology, topography, and chemical and physical characteristics similar to tissues, particularly to generate a 3D scaffold for neuronal growth.^213–215^ Due to the vastness of this topic and scope of this review, we refer readers to more dedicated reviews for more information.^19,98,103,185–190^

### D. Magnetic field

**FIG. 15.**
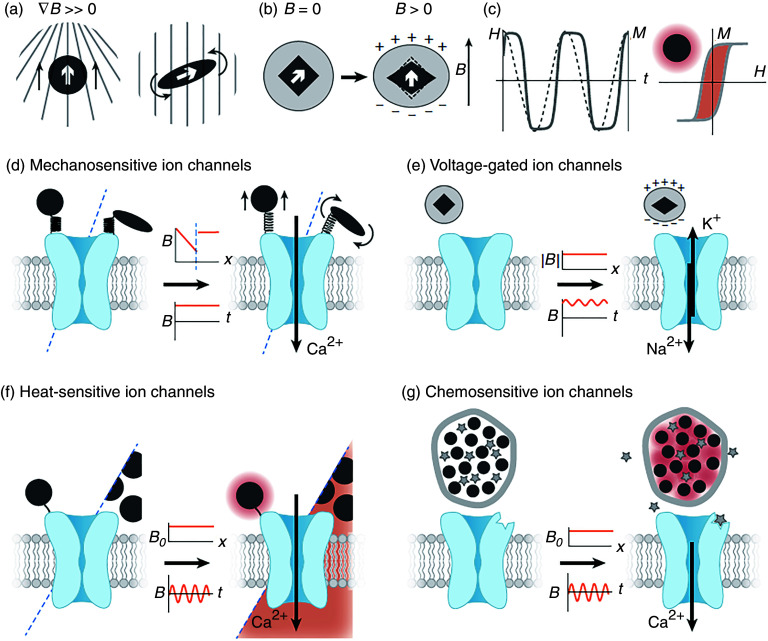
(a) A magnetic nanoparticle can be used to apply a force or torque. To be able to generate torque, the magnetic particle should be asymmetric. (b) Magnetoelectricity is the property of a material through which it can be polarized when a coupling between magnetic and electric fields is established. (c) Loops of hysteresis can be used to generate a thermal response. (d) A neuron's mechanosensitive ion channel can be modulated through the generation of force or torque using a magnetic nanoparticle. (e) A magnetoelectric nanoparticle can be polarized remotely to control voltage-gated channels. (f) Magnetothermal properties can be used to modulate the behavior of heat-sensitive channels. (g) Magnetic nanoparticles can be attached with moieties that release themselves under the magnetic field to influence chemosensitive ion channels. (a)–(g): *B* is the magnetic flux density, *M* is the magnetization, and *H* is the magnetic field strength. Whereas, *B* vs *x* shows the spatial variation of magnetic field, and *B* vs *t* shows the change in magnetic field as a function of time. Reprinted with permission from M. G. Christiansen *et al.*, Annu. Rev. Neurosci. **42**, 271–293 (2019). Copyright 2019 Annual Reviews.^216^

A magnetic field is one of the noninvasive techniques that can be used to control neurons *in vitro*. Although the magnetic field has a wide range of applications in neuroscience, here, we will only discuss those related to the use of magnetic nanomaterials to influence neuronal growth. Because of their remote controllability, small size (a few nanometers to a few hundred nanometers), and high biocompatibility, magnetic nanoparticles make an ideal candidate for high spatiotemporal and dynamic control of neuron growth as well as neuronal activity. Some of the possible ways of using magnetic nanoparticles for neuronal growth control are depicted in Fig. 15. When put in a nonuniform magnetic field, a magnetic particle aligns itself toward a high magnetic field direction and applies a positive force if energy gained due to magnetic movement is higher than the thermal fluctuations.^216^ This force has been used to control axon growth rate as well as guidance.^181–184^

In another set of experiments, researchers have shown that an anisotropic magnetic nanoparticle may also overturn or swing due to torque generation under the magnetic field with low frequency and influence the cell behavior through selective activation of mechanosensitive or chemosensitive channels.^180,217–219^ For example, the thermal energy can be generated near or around cells using a magnetic nanoparticle by alternating rotating or spinning of anisotropic magnetic nanoparticles under a changing magnetic field or through energy loss during the hysteresis loop, which in turn can be used to modulate neuronal growth.^220,221^ Besides, magnetoelectric materials are a special kind of composite material that can be polarized by the coupling of a magnetic field and an electric field.^178,179^ Given that cells are highly responsive to changes in the electric field around them, these magnetoelectric nanomaterials provide comfortable remote control over the behavior of neurons.^222,223^ Moreover, if the particles are <20 nm, they can also be applied to cross the blood–brain barrier to stimulate the neurons inside the brain.^223^

### E. Electric field and conductivity

The notion that neuron growth can be controlled using voltage gradients (i.e., electric field) is very old.^192,193,224^ As early as 1920, Ingvar reported that neuron cultures of fibers from explanted chick brain tissue behaved differently at the anode and cathode when a DC of extremely low strength was applied.^224^ According to him (no supporting figures were present), the outgrowth of cells and fibers was confined entirely along the line of the galvanic field. There was also a morphological difference in the neurons growing at the anode and cathode. Although Ingvar's results remained highly debated and irreproducible, in 1946, Marsh and Beams^225^ were able to show convincingly that neuronal growth responds to electric current. However, their results were very different from Ingvar's, and they did not see morphological differences between neurons growing at the cathode and anode.^224,225^ Also, they showed that at only >100 *μ*A/mm^2^ of electric current density (calculated for the cross-sectional area of the medium), neurites of chick medullary explants grew away from the anode, and at 120 *μ*A/mm^2^, the neurites turned toward the cathode.^225^ Since then, cathode-oriented growth of neurons has been confirmed and validated by several other researchers.^191–195^ Nonetheless, a complete mechanism of the electric field–induced orientation of the growth cone is not clear. Some experiments suggest that because most proteins have a net negative charge at physiological pH when a constant extracellular voltage is applied in the medium, these proteins tend to redistribute through electrophoresis or electroosmosis to create an asymmetry similar to chemotropic gradients to influence growth cone guidance.^226,227^ However, it is important to note that the attraction of the growth cone toward a cathode is not universal, and whether it will be repelled, attracted, or remain static depends on a wide range of factors, including nerve cell type, the substrate, and its adhesion, and whether the neurite process is axonal or dendritic.^228^

**FIG. 16.**
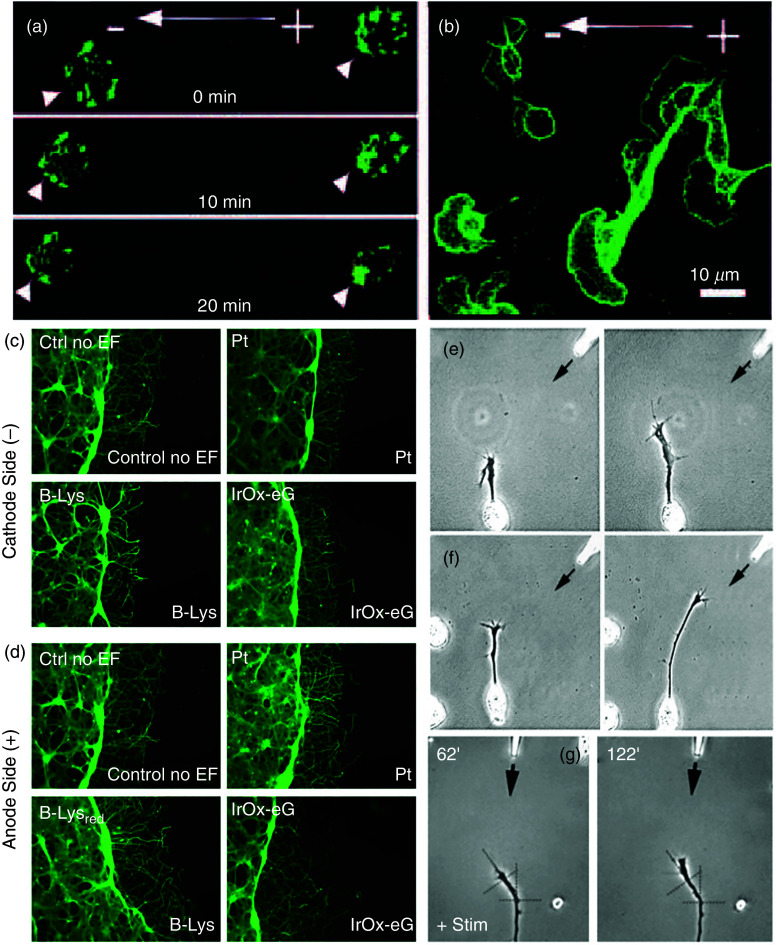
(a) and (b) An example of the redistribution of growth factors under an electric field. Shown is the redistribution of epidermal growth factor (EGF) receptors to the cathode side of migrating corneal epithelial cells, thereby suggesting that the asymmetric distribution of growth factors in the presence of an electric field may be instrumental in the electric field–directed guidance of axons. Reprinted with permission from C. D. McCaig *et al.*, Physiol. Rev. **85**, 943–978 (2005). Copyright 2005 The American Physiological Society.^228^ (c) and (d) The spontaneous neurite outgrowth of scratched cortical neuron cultures on the cathode and anode, respectively, for different electrode materials in the presence of an electric field. It is evident that electroactive materials, such as bilayers of poly(3,4-ethylenedioxythiophene) (PEDOT) and polypyrrole with lysine counterions (B-Lys) or iridium oxide with pristine graphene hybrids (IrOx-eG), promote higher regeneration in the scratched region compared to only platinum electrode or control. EF denotes electric field. Reprinted with permission from M. P. Lichtenstein *et al.*, Appl. Mater. Today **6**, 29–43 (2017). Copyright 2016 Elsevier.^230^ (e)–(g) Growth cone micrographs at the start (right) and end (end) of exposure to a gradient of growth factor. (e) The growth cones of young *Xenopus* neurons (6–10 h in cultures) are repelled when they are exposed to netrin-1's gradient of 5 *μ*g/ml through a pipette. (f) However, if the same neurons were electrically stimulated before netrin-1 exposure, the growth cones responded attractively to exposure to a similar gradient of netrin-1. (g) The repulsive behavior of rMAG can be transformed into an attractive response if the neurons are electrically stimulated before rMAG exposure. Reprinted with permission from G.l. Ming *et al.*, Neuron **29**, 441–452 (2001). Copyright 2016 Elsevier.^199^

Moreover, other than simple neuronal guidance, the electric field can also increase the outgrowth of neurons toward cathodes. For example, Patel and Poo showed that the neuron outgrowth is significantly enhanced toward the cathode in the presence of an electric field.^195^ Similarly, McCaig *et al.* showed that the neurons preferentially outgrow toward the cathode, while they are retracted or reabsorbed at the anode.^229^ Also, McCaig *et al.* were able to induce regrowth in ∼47% of reabsorbed neurons by reversing the polarity. This later inspired them to use an alternating DC electric field for the repair of adult mammalian spinal cord lesions.^226^ In another work, Lichtenstein *et al.* showed that large-charge-capacity nanostructured electrodes after short-term electrostimulation could enhance neural repair *in vitro*^230^ [Figs. 16(c) and 16(d)]. Moreover, in addition to neuronal outgrowth, the applied DC electric field has also been shown to enhance the branching of neurons.^196–199^ In some studies, the AC electric field has been proposed as a better alternative to the DC electric field because the AC electric field can limit the risk of electrolysis at the electrodes.^231^

In 1998, Catalano and Shatz showed that neuronal electrical activity was essential for axon pathfinding and target selection in thalamic axons.^232^ This provided the basis for another way to influence *in vitro* neuronal growth by inducing neuronal activity through electrical stimulation. Several experiments have demonstrated that electronically induced neuronal activity can be used to modulate and control neuronal growth *in vitro*.^233^ For example, Ming *et al.* were able to influence the behavior of growth guidance factors such as netrin-1 and myelin-associated glycoprotein (MAG) using electrical stimulation^199^ [Figs. 16(c)–16(e)]. Their results demonstrated that depending on whether a culture of spinal neurons was young (<10 h in culture) or old (>16 h in culture), the response to the netrin-1 gradient can, respectively, be repulsive or attractive. However, if the same young cultures of spinal neurons were exposed to a short duration of electrical stimulation, the repulsive response of netrin-1 changed to an attractive response. Additionally, a short course of electrical stimulation on the older cultures produced an increased attraction to netrin-1.^199^ Similarly, Ming *et al.* also found that although recombinant MAG (rMAG) caused a repulsive turn for the growth cone of older cultures; after an external electrical stimulation, the repulsive turning changed to attractive.

Although electric field application has immense potential for application in control of neuronal growth, before it can be applied for any useful application, a more concentrated effort is required to understand how the electric field influences neurons, especially in terms of electrochemical changes at the cellular level. In Sec. IV F, we discuss the optical control of neuronal growth.

### F. Optics

**FIG. 17.**
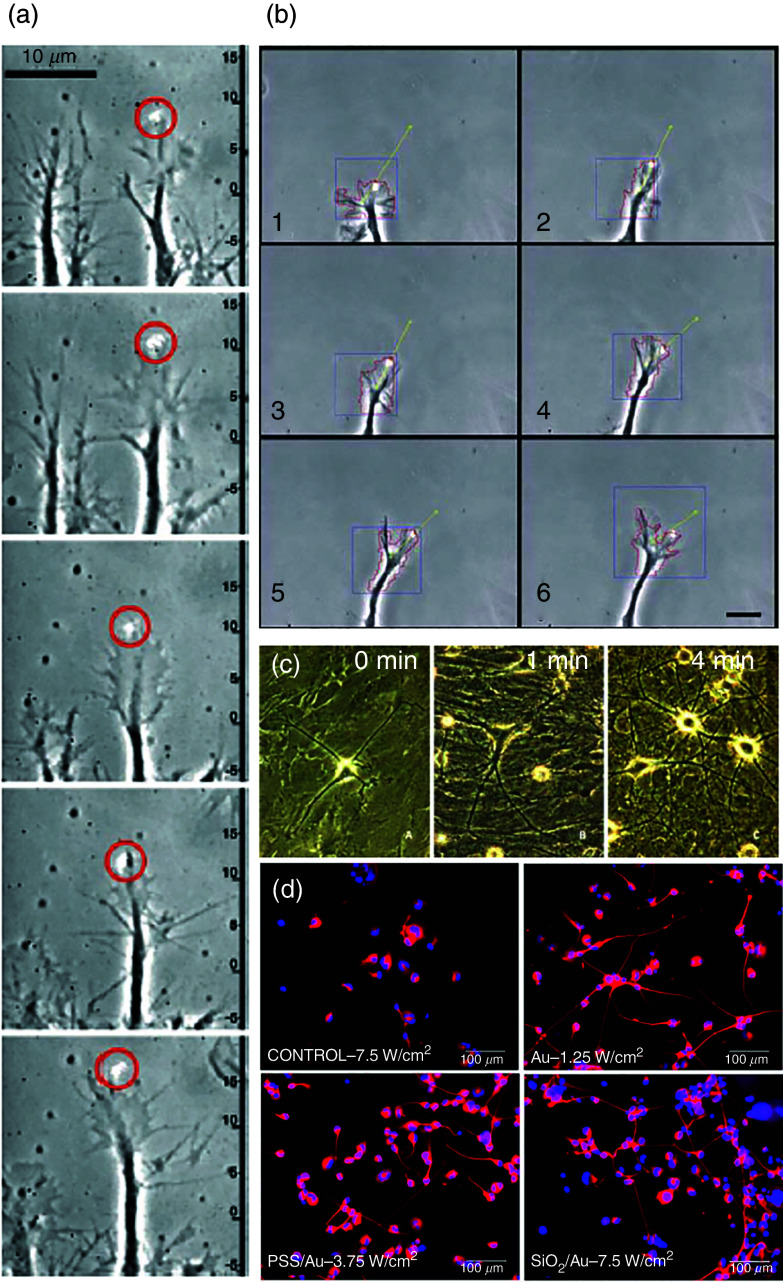
(a) Neuronal outgrowth with and without laser illumination. It is evident that the growth cone under laser illumination (laser spot marked by a red circle) shows greater extension in the presence of a 20-mW laser spot. From top to bottom, each photo was taken at a 10-min interval. Reprinted with permission from A. Ehrlicher *et al.*, Proc. Natl. Acad. Sci. U.S.A. **99**, 16024 (2002). Copyright 2002 The National Academy of Sciences.^238^ (b) Automatic control of the growth cone using a line profile created and controlled by a computer-controlled spatial light modulator. Left to right (1–6), each photo was taken 2 min apart. Reprinted with permission from D. J. Carnegie *et al.*, J. Biophotonics **2**, 682–692 (2009). Copyright 2009 Wiley‐VCH.^239^ (c) Comparative microscopic image of nerve cell body size and neuronal branching in control cells and cells illuminated with the laser for 1 and 4 min, respectively. It is evident that the branching in neuronal cultures illuminated with the laser is significantly high compared to control. Also, those illuminated with the laser have thicker branches (original magnification ×300). Reprinted with permission from S. Rochkind *et al.*, Lasers Surg. Med. **41**, 277–281 (2009). Copyright 2009 Wiley‐Liss.^240^ (d) The photos show the effect of laser irradiation on NG108-15 cells without Au nanorods, with Au nanorods, with Au nanorods coated with PSS, and with Au nanorods coated with SiO_2_. It is evident that cells with Au nanorods showed maximum neurite length. Also, the neurite length was not dependent on the surface of Au nanorods, as both PSS/Au and SiO_2_/Au samples showed similar neurite length compared to bare nanorods. Reprinted with permission from C. Paviolo *et al.*, Biotechnol. Bioeng. **110**, 2277–2291 (2013). Copyright 2013 Wiley Periodicals.^245^

Photobiomodulation [low-level laser (light) therapy] and its application to neurology and neuroscience are not new and have been known for several decades.^234^ Although a complete photobiomodulation mechanism is not yet clear, it has already found its usage as a potential therapy for traumatic brain injury.^234–236^ A detailed discussion on photobiomodulation is out of the scope of this review, and readers should refer to other excellent reviews on the subject.^234,235,237^ Here, we limit our discussion to a part of photobiomodulation that concerns the control of *in vitro* neuronal growth using light. Perhaps the first direct evidence of control of *in vitro* neuronal growth using light was provided by Ehrlicher *et al.*^238^ Using both rat and mouse neuronal cell lines, they showed that the optical effect on neuronal growth is highly robust and can successfully be reproduced for a broad range of laser powers.^238^ They were able to guide and bifurcate the neuron growth cone using light and showed that light exposure could also enhance the growth rate of a neuron [Fig. 17(a)]. Their analysis showed that the heating due to the laser was very limited, and there was no optical trap effect (i.e., there was no mechanical force),^238^ which led them to conclude that the outcome of their experiments must be a result of increased polymerization of actin monomers, which drives the growth cone in the direction of light and enhances the neuron growth rate. In another report, Carnegie *et al.* designed a computer-controlled spatial light modulator (SLM) [Fig. 17(b)] to steer and manipulate the shape of a laser beam to guide the neuronal growth.^239^ Similarly, Rochkind *et al.* were able to show that embryonic rat brain culture showed accelerated fiber sprouting and neuronal cell migration when exposed to 780-nm laser irradiation for more than 1 min^240^ [Fig. 17(c)]. In contrast, Higuchi *et al.* were able to both suppress and enhance neuronal growth by modulating the wavelength and power of visible lasers.^241,242^ Ebbesen and Bruss proposed that laser-induced effects on neuronal growth may arise due to localized heating (instead of light-induced polymerization).^243^ The work of Ebbesen and Bruss was further substantiated by Oyama *et al.*, who were able to achieve a very high neurite elongation rate (>10 *μ*m/min) using a microheater platform.^244^ For local microheating, they used a focused laser beam under a microscope. Through a detailed investigation, Oyama *et al.* concluded that the increase in neurite growth rate due to localized (microscopic) heating is due to the activation of microtubule polymerization and microtubule sliding.^244^ In yet another example, Paviolo *et al.* were able to control the neuronal branching through plasmonic-assisted heating.^245^ They showed that NG108–15 cells did not show any enhancement in neuronal branching or outgrowth after irradiation with a laser of wavelength 780 nm and a power of 1.2–7.5 W/cm^2^. However, after the uptake of Au nanorods, when these cells were illuminated with the same power laser, they showed a higher level of branching and outgrowth [Fig. 17(d)]. Additionally, to show that the neuronal branching was not an effect of the nanorod surface, they coated the Au nanorod surface with poly(4-styrene sulfonic acid) (PSS) and SiO_2_ and did not see any difference in neuronal outgrowth or branching after laser irradiation [Fig. 17(d)]. Obviously, more investigations are needed, but at this point, it seems that laser irradiation leads to localized heating, which in turn initiates actin and microtubule polymerization. The increased actin polymerization reduces the retrograde flow and induces the pushing forces within the growth cone for forwarding movement, while the microtubule polymerization provides an impetus for the outgrowth of neurons.

## V. FUTURE OUTLOOK

Our review provides an overall background on the various biological processes involved in the growth of neural networks *in vitro*. It is clear that these biochemical and biophysical processes are not independent of each other but play a complementary role in formulating the underlying mechanisms of neuronal growth. Furthermore, the understanding of neuronal growth in response to physical and chemical cues from the surrounding environment has opened up possibilities for controlling and engineering neuronal growth for various applications in biomedical engineering. Nonetheless, there are still several open questions pertaining to better understanding neuronal growth. At the same time, there is a lot of untapped potential for applications of what we already know about neuronal growth until now. In this section, we present our perspective on some of the open questions and opportunities in this regard. We believe that four areas require specific consideration.

### A. Materials and substrates

The mechanisms behind physical, chemical, mechanical, or electronic modulation of neuronal growth remain highly debated. Hence, there is a requirement for designing substrates from biocompatible materials, which can support neuronal growth while also providing the ability to study these parameters. While a lot of materials and substrates have been used for neuronal growth, part of the issue in understanding mechanisms behind neuronal growth is that there is a mismatch between the requirements of biological tissues with the available techniques, especially *in vivo*. For instance, electronic modulation mainly utilizes hard/sharp metal electrodes that suffer from modulus mismatch and chemical corrosion in biological environments, hence adversely affecting the extracellular matrix. Therefore, in addition to biocompatibility, the chemical, physical, and electrical properties of the substrates need to be better designed for understanding their influence and role in engineering neuronal growth. In fact, modulation of substrate properties provides an opportunity to create gradients in chemical composition, electrical conductivity, and forces to engineer neuronal growth as well as to understand the biochemical and biophysical mechanisms involved in neuronal network formation. For example:

• The mechanisms behind stretch-induced neuronal growth and tension-induced polarization in neurons are still not understood. While mechanotransduction is thought to be involved in these processes, its precise role in neuronal polarization in response to force or tension is not understood. In this regard, flexible substrates can be used to introduce various forces to study the effect of stress and tension on neuronal growth.^246,247^
• Materials such as hydrogels are known to better mimic the brain's extracellular matrix compared to inorganic substrates due to their soft and biocompatible nature.^185^ Electrically conductive hydrogels are one of the recent material systems being tested for neural tissue engineering.^248,249^ Having electrical cues along a biomimicking environment provides a better substrate system to study electrical field effects and modulate neuronal networks compared to conventional substrates like glass.
• To understand the extent of how extrinsic or intrinsic growth factors influence neuronal growth, substrates can be designed to incorporate and have a controlled release of specific growth factors, and neuronal growth can be studied in response to their release.^250^

### B. 3D model systems

Most of the studies for neuronal growth in response to physicochemical cues are done *in vitro* on 2D substrates. While this simplifies the system, it limits the scaling of the proposed mechanisms *in vivo*, as the real tissues, which are a three-dimensional environment, cannot be entirely and accurately mimicked. For neuronal systems, brain organoids have emerged as a current-generation 3D model system to overcome the fundamental limitations of 2D systems. Working with brain organoids will enable understanding of various mechanisms in 3D while also providing a better model system to use optical, physical, chemical, and electrical stimulation in 3D to understand better several aspects of neuronal growth and engineering.^251–253^

### C. Recording and modulation techniques

Given the complexity and variety of neuronal systems, we need *in vitro* systems that can run many experiments in parallel while also having a very high resolution of the output. The latest developments in high-density multielectrode arrays are impressive. Various groups, including ours, are now focusing on developing nanometer-sized recording electrodes as a step forward to provide better spatial resolution.^254–256^ At the same time, multiwell systems with high pixel output are needed to achieve single-neuron resolution as well as to allow simultaneous measurements on multiple cells.^257,258^ Such systems can also be combined with different techniques to measure real-time changes in the chemical or physical environment of a neuron.

Magnetic nanoparticles may be the next step for large-scale neuronal growth control and studying cellular interconnection mainly because they provide noninvasive, wireless control over neurons.^216^

Also in the future, combining two or more external cues or modulation techniques for more advanced control of neuronal growth will be interesting. That is, one could explore the possibility of doing programmable neuron growth by combining either light, electrical, magnetic, or mechanical stimulation. This will also provide an opportunity to untangle the effects of one technique over another.

### D. Data and artificial intelligence

Neuronal studies result in complex and large datasets. It is becoming imperative to use new techniques in deep learning and artificial intelligence to understand neuronal growth, signaling, and mechanisms better.

Most importantly, given the extreme complexity and limited understanding of neuronal growth and function, interdisciplinarity and collaboration among engineers, physicists, chemists, nanotechnologists, computer scientists, stem cell biologists, and neurobiologists have become the need of the hour to overcome the challenges.

### E. Concluding remarks

Research in neuroscience and especially neuroengineering is becoming increasingly interdisciplinary, and it is often difficult for beginners from different backgrounds to develop a comprehensive overview of the basics of neuronal growth. Therefore, our goal in this review has been to unify different aspects of neuronal growth under a common framework such that it is acceptable to both established and new researchers from a wide range of disciplines. It should be noted that most biochemical and biophysical concepts presented in this review are based on studies of neuronal growth and development *in vitro*, which differ in specific ways from *in vivo* nervous systems. This limitation is expected to propagate to our understanding of the mechanisms and the technological applications listed here. This review is a quick reference guide for established researchers on several of the budding research topics related to neuronal growth. At the same time, for new researchers, this review is an essential academic source to develop an understanding of the principles behind neuronal growth and neuroengineering. Moreover, we expect that our perspective on future approaches for engineering and modulating neural networks will provide the pathway for further advancement in the field.

## Data Availability

Data sharing is not applicable to this article as no new data were created or analyzed in this study.
